# Defects in intron recycling suppress the antiviral response via a mechanism of intronic endogenous dsRNA

**DOI:** 10.1084/jem.20250344

**Published:** 2026-03-12

**Authors:** Chaorui Duan, Luke Buerer, Cory Bowers, Allison J. Taggart, Mara H. O’Brien, Sarah Gunasekera, Chien-Ling Lin, Jing Wang, Yi Zeng, Jonathan P. Staley, Alger M. Fredericks, Sean F. Monaghan, Anastasia Welch, Nathaniel E. Clark, Daxing Gao, Nico Marr, Shen-Ying Zhang, Jean-Laurent Casanova, William G. Fairbrother

**Affiliations:** 1 Department of Molecular Biology, Cell Biology, and Biochemistry, Brown University, Providence, RI, USA; 2 Center for Computational Molecular Biology, Brown University, Providence, RI, USA; 3 Giuliani RNA Center, Brown University, Providence, RI, USA; 4 Institute of Molecular Biology, Academia Sinica, Taipei, Taiwan; 5 Department of Molecular Genetics and Cell Biology, University of Chicago, Chicago, IL, USA; 6 Department of Surgery, The Miriam Hospital, Providence, RI, USA; 7 Division of Surgical Research, Department of Surgery, Alpert Medical School of Brown University/Rhode Island Hospital, Providence, RI, USA; 8 Department of General Surgery, The First Affiliated Hospital of USTC, Division of Life Science and Medicine, University of Science and Technology of China, Hefei, China; 9 Institute of Immunology and the CAS Key Laboratory of Innate Immunity and Chronic Disease, Division of Life Sciences and Medicine, University of Science and Technology of China, Hefei, China; 10 St. Giles Laboratory of Human Genetics of Infectious Diseases, Rockefeller Branch, The Rockefeller University, New York, NY, USA; 11 Department of Human Immunology, Sidra Medicine, Doha, Qatar; 12 Laboratory of Human Genetics of Infectious Diseases, Necker Branch, INSERM U1163, Paris, France; 13 Paris Cité University, Imagine Institute, Paris, France; 14 Howard Hughes Medical Institute, New York, NY, USA; 15 Pediatric Immunology-Hematology Unit, Necker Hospital for Sick Children, Paris, France

## Abstract

Loss of the lariat debranching enzyme DBR1 causes cytoplasmic accumulation of intron lariats, but why this reduces cell-intrinsic immunity is unclear. Here, we show that intronic inverted repeats Alu (IR Alus), normally degraded after splicing, form long double-stranded RNA (dsRNA) structures when lariats escape recycling. Viral introns evolve under pressure to avoid dsRNA, whereas human introns are enriched for them. Using computational, immunostaining, and genomic approaches, we demonstrate that DBR1 deficiency elevates cytoplasmic dsRNA and attenuates RNase L and PKR signaling. Our data suggest high levels of IR Alu dsRNA titrate PKR, potentially providing a mechanistic explanation for viral susceptibility in DBR1-deficient cells. Cytoplasmic RIP-seq against dsRNA finds introns to be a more abundant source of IR Alus than 3′ UTRs in WT cells. Our findings suggest the high load of IR Alus in introns creates a situation where the efficiency of lariat recycling is a powerful modulator of endogenous dsRNA levels in human cells.

## Teaser

Defective intron recycling leads to the accumulation of intron-derived double-stranded RNA in the cytoplasm, which suppresses PKR and RNase L activation. This work reveals how intronic Alu repeats and RNA metabolism shape endogenous dsRNA levels and cell-intrinsic immunity.

## Introduction

The excision of introns from pre-mRNA is an essential step in the production of mature mRNA transcripts. Intron removal is catalyzed by the spliceosome through two sequential nucleophilic attacks by sugar hydroxides on the phosphates in a phosphodiester bond that connects introns to exons (Wahl et al., 2009; Wilkinson et al., 2020). The first attack separates the upstream exon from the 5′ end of the intron, which forms a 2′–5′ linkage at the branchpoint to create the lariat intermediate. The second step involves a nucleophilic attack by the free hydroxyl of the upstream exon on the downstream 3′ splice site, resulting in exon ligation and the excision of the intron lariat (Padgett et al., 1984; Ruskin and Green, 1985; Ruskin et al., 1984). The vast majority of lariats are quickly linearized by the lariat debranching enzyme DBR1, which hydrolyzes the 2′–5′ branchpoint bond (Chapman and Boeke, 1991; Montemayor et al., 2014; Ruskin and Green, 1985). After debranching, intron lariats undergo further digestion into small RNAs or free nucleotides available for the next cycle of transcription. In addition, lariat RNAs and their branchpoints can be sequenced and characterized using specialized approaches, including Shapeshifter, lariat sequencing, and co-transcriptional lariat sequencing (Awan et al., 2013; Mercer et al., 2015; Pineda and Bradley, 2018; Taggart et al., 2012; Taggart and Fairbrother, 2018; Zeng et al., 2022).

Studies in mice and humans have revealed a range of pathologies caused by the loss of DBR1 activity (Findlay et al., 2014; Zheng et al., 2015). Deficiencies in human DBR1 may lead to conditions such as cancer or impact hinder HIV replication (Galvis et al., 2014; Han et al., 2017; Xu et al., 2022; Ye et al., 2005). Homozygous *DBR1* variants lead to the accumulation of RNA lariats and are associated with a congenital ichthyosis-like phenotype (Shamseldin et al., 2023). Moreover, biallelic severely hypomorphic *DBR1* variants confer increased susceptibility to viral encephalitis due to herpes simplex virus 1 (HSV-1) infection. Elevated lariat levels were observed upon HSV-1 infection in WT cells, which were enhanced by DBR1 deficiency in the host (Zhang et al., 2018). More recent, Chan et al. found that a homozygous pathogenic mutation in *DBR1* (DBR1 I120T/I120T) resulted in the accumulation of RNA lariats, which impaired antiviral immune responses and allowed uncontrolled SARS-CoV-2 replication in hindbrain neurons, leading to severe encephalitis (Chan et al., 2024; Valeri and Kajaste-Rudnitski, 2025). These findings suggest that defects in lariat metabolism in the absence of DBR1 are linked to impaired cell-intrinsic immunity.

However, the specific mechanisms by which lariats modulate antiviral response have yet to be elucidated. The best-characterized antiviral RNA-sensing pathways detect cytosolic double-stranded RNA (dsRNA). For example, MDA5 and RIG-I recognize viral dsRNA and trigger signaling cascades that lead to the production of interferons and pro-inflammatory cytokines, initiating an antiviral immune response (Chen and Hur, 2022; Hur, 2019). The OAS/RNase L pathway, activated by interferon signaling, degrades both viral and host RNA, promoting apoptosis and inhibiting viral replication (Cooper et al., 2014; Zhou et al., 1997). Similarly, PKR, another dsRNA sensor, phosphorylates the eukaryotic initiation factor eIF2α, resulting in global shutdown of protein synthesis and inhibition of viral replication (García et al., 2006; Hur, 2019). One of the challenges dsRNA sensors face is making a distinction between pathogenic signal and the low levels of endogenous dsRNA expressed during normal cellular function (Schlee and Hartmann, 2016). To achieve this, these sensors employ distinct physical criteria that collectively integrate duplex length, structure, and concentration. A key determinant is duplex length. OAS1 requires stems of at least 17 bp to produce 2′–5′ oligoadenylates (Calderon and Conn, 2018; Vachon et al., 2015), while PKR is activated by duplexes of ∼33 bp, reflecting the requirement for dimerization and interdimeric phosphorylation (Chen and Hur, 2022; Zheng and Bevilacqua, 2004). RIG-I preferentially recognizes short to intermediate duplexes (22–500 bp), with additional sensitivity to 5′-triphosphate or diphosphate ends, whereas MDA5 requires long continuous stems of 0.5–1 kb or more to assemble extended filaments along the RNA backbone (Chen and Hur, 2022). Structural integrity further modulates recognition, as bulges, mismatches, and internal loops attenuate activation, particularly for PKR and OAS, which depend on helical continuity (Calderon and Conn, 2018; Heinicke et al., 2011). Finally, maximal response depends on an optimal stoichiometry of dsRNA to dsRNA sensor concentration. For PKR, this property creates a characteristic “bell-shaped” activation profile with low concentrations of dsRNA stimulating activity until a peak and then higher concentrations reducing activity. This behavior has been explained in previous in vitro studies by a model in which low dsRNA concentrations allow PKR to dimerize on the same RNA, whereas high dsRNA levels redistribute PKR onto excess RNA, dispersing dimers into monomers and thereby limiting activation (Galabru and Hovanessian, 1987; Katze et al., 1991; Lemaire et al., 2008; Williams et al., 1979). Given PKR’s central role in antiviral defense, its loss or functional impairment increases susceptibility to infection, including influenza and HSV-1, by allowing enhanced viral replication and, in some cases, severe outcomes, as observed with vesicular stomatitis virus (Balachandran et al., 2000; Khabar et al., 2000).

In this study, we tested the hypothesis that the dsRNA-sensing antiviral response is impaired by the stable lariat introns that accumulate in DBR1-deficient cells. We analyzed both viral and host introns, finding intronic dsRNA structure to be underrepresented in viruses and overrepresented in humans. The dominant source of secondary structure in human introns comes from inverted repeat (IR) Alu elements. In the absence of DBR1 activity, we visualized lariat accumulation in the cytoplasm and an increase of cytoplasmic dsRNA. There is also a loss of RNase L activity and lower levels of PKR and eIF2α phosphorylation in DBR1-depleted cells, which can be rescued by the add-back of DBR1. We characterized the sequence diversity of IR Alus and define how the frequent bulges and mismatches break otherwise long duplex stems into arrays of shorter dsRNA stretches. Finally, we proposed a titration model (similar to the in vitro model) to explain these findings. We suggest cellular PKR that could dimerize on dsRNA is dispersed as monomers across separate dsRNA molecules in the presence of an excess of dsRNA.

## Results

### Multiple viruses inhibit intron turnover in cells with DBR1 deficiency

**Figure S1.**
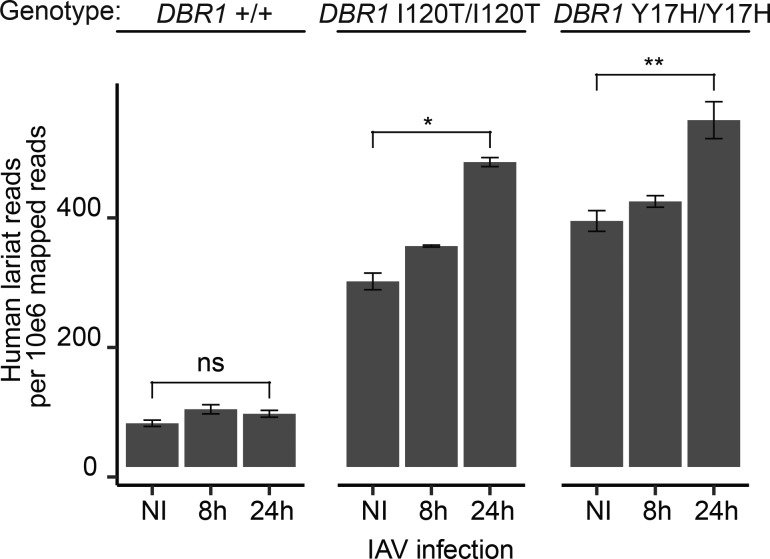
**Cellular lariats increase in *DBR1* mutant cells upon influenza virus infection.** Human lariat levels in RNA-seq data from WT (*DBR1 +/+*) and *DBR1* mutant (*DBR1* I120T/I120T and *DBR1* Y17H/Y17H) patient cell lines that were not infected (NI), infected with influenza virus (IAV) for 8 h, and infected with IAV for 24 h (P value from two-sided *t* test; ns, not significant; *P < 0.05, **P < 0.01).

Individuals with severely hypomorphic biallelic *DBR1* gene variants can be vulnerable to life-threatening brainstem lesions when exposed to HSV-1, norovirus, or other viruses (Zhang et al., 2018). Previous research demonstrated that while HSV-1 infection leads to increased lariat levels in cells derived from both healthy controls and *DBR1* mutant patients, the lariat increase is significantly larger in *DBR1* mutant cells. Here, we utilize influenza virus to infect fibroblasts derived from control and *DBR1* mutant patients (DBR1 *I120T/I120T* and DBR1 *Y17H/Y17H*) to confirm that this connection between virus infection and decreased intron recycling can be extended to other viruses. We observed significant 41% (I201T) and 64% (Y17H) increases in human lariats postinfluenza infection in *DBR1* mutant patient cell lines (Fig. S1). As the influenza and HSV-1 genomes are both composed of intron-containing genes, it is possible that some feature of viral introns could dominantly inhibit human lariat turnover. To test this hypothesis, we performed sequence analysis on the set of human introns and all annotated introns from viruses known to infect humans.

### Viral introns evolve under strong selective pressure against RNA secondary structure

**Figure 1.**
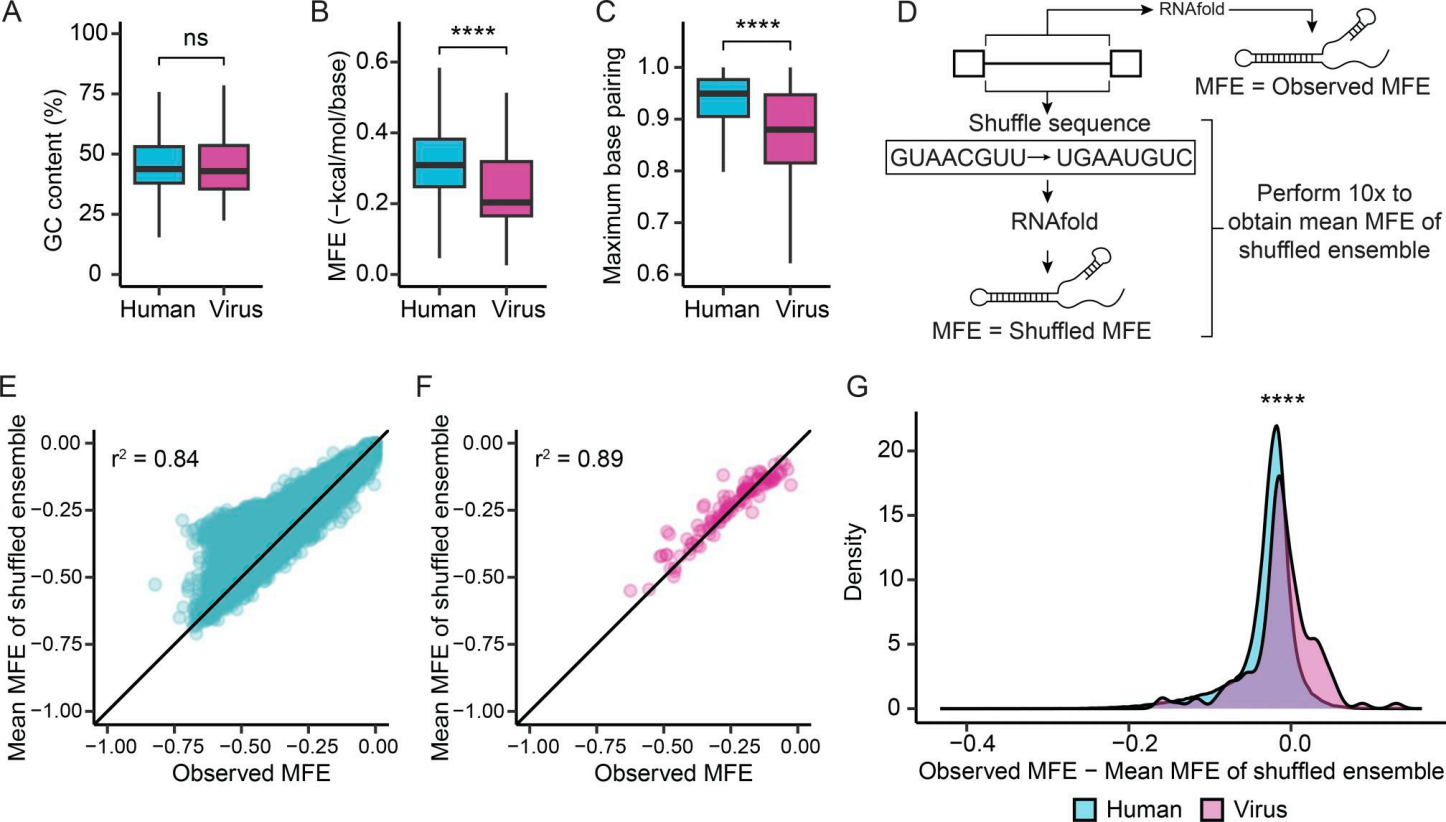
**Viral introns are less structured than 90% of human introns. (A–C)** Human introns (length <5,000 nt) compared with 126 introns from human viruses according to their distributions of (A) GC content, (B) normalized predicted MFE intron length ≤5,000 nt), and (C) potential for intron base pairing (MBP, see Materials and methods). P values were from *t* test. ns, not significant; ****P < 0.0001. **(D)** Schematic describing computational experiments comparing the observed predicted MFE of an intron to the expected value (predicted MFE of an ensemble of 10 shuffles of the same intron). **(E)** For human introns, each intron’s observed MFE is plotted against the mean MFE derived from the intron’s shuffled ensemble described in D. **(F)** For human virus introns, each intron’s observed MFE is plotted against the mean MFE derived from the intron’s shuffled ensemble described in D. **(G)** The distribution of the difference between observed MFE and mean MFE of shuffled ensemble for human and viral introns. P value was from *t* test. ****P < 0.0001.

**Figure S2.**
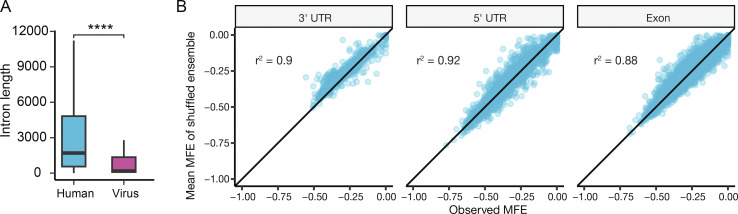
**Comparison of viral and human intron lengths and secondary structure in human transcripts. (A)** Viral introns are shorter than human introns. P value was from *t* test, ****P < 0.0001. **(B)** Transcript regions exported to the cytoplasm possess secondary structure in line with random expectation. For human 3′ UTRs, 5′ UTRs, and exons, each region’s observed MFE is plotted against the mean MFE derived from the region’s shuffled ensemble described in Fig. 1 D.

We downloaded the sequences of 43 human-tropic viruses and their 126 introns from the National Center for Biotechnology Information (NCBI) Virus database (Hatcher et al., 2017). While GC content does not significantly differ between human and viral introns (Fig. 1 A), viral introns exhibit lower levels of predicted structure. On average, viral introns are shorter than human introns (Fig. S2 A, 9.3-fold, P = 7.08e−154). However, even after normalizing for length, minimum free energy (MFE) values from RNA-folding predictions are strikingly higher for viral introns, with the median viral intron MFE falling in the lower 10% of stability in the human intron MFE distribution (Fig. 1 B, P = 1.09e−09). While this result implies selection against secondary structure, we sought a more robust measure that did not rely on structure prediction. We developed a maximum base pairing (MBP) metric that measured the maximum proportion of positions that could be paired. MBP potential equals 1.0 when complementary bases occur with equal frequency in the sequence (accounting for G-U wobble base pairing; see Materials and methods). The median viral intron has 7% lower MBP potential than the median human intron, suggesting the possibility that viral sequences utilize nucleic acids in an unbalanced combination that would ensure unpaired bases and therefore less secondary structure formation (Fig. 1 C, P = 2.12e−10). We further interrogated the difference between the observed structure of an intron and the structure expected based on sequence composition. To do this, we generated an ensemble of 10 shuffled intron sequences for each intron and calculated the mean MFE of this shuffled ensemble (Fig. 1 D). Deviations between the observed and shuffled MFEs indicate introns with more or less structure than expected from their nucleotide composition. The distribution of observed and shuffled MFEs for human introns indicates that, while there is often agreement between these values, there are subsets of introns for which the observed MFE is much lower than the mean shuffled MFE (Fig. 1 E). In contrast, similar experiments for viral introns resulted in little difference between the observed MFEs and the expectation based on the ensemble of shuffled sequences (Fig. 1, F and G). These results suggest that viral introns face selective pressure toward less secondary structure. This trend was also observed for 5′ untranslated regions (UTRs), exons, and 3′ UTRs, which, similar to viral introns, are adapted to exist at high levels in the cytoplasm (Fig. S2 B).

### IR Alu elements contribute to human intron structure

**Figure 2.**
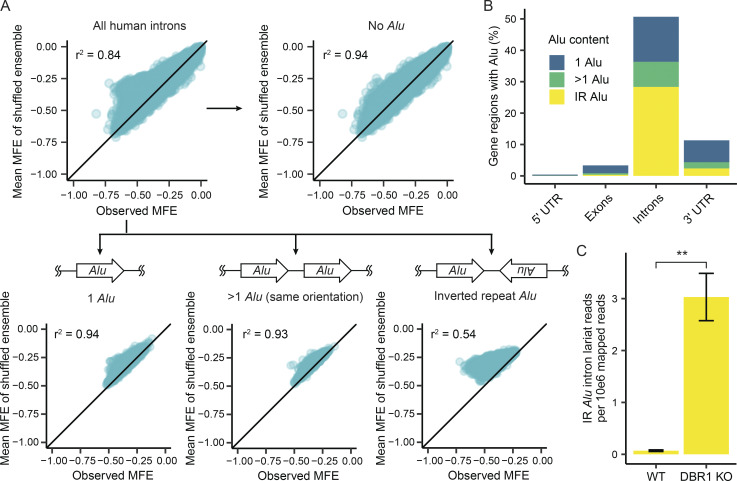
**IR *Alu* elements contribute to human intron structure. (A)** The relationship between observed MFE and the mean MFE of an ensemble of shuffled intron sequences for human introns is partitioned into distinct categories based on each intron’s Alu content (no Alu, 1 Alu, >1 Alu in the same orientation, and IR Alu). **(B)** The proportion of 5′ UTR, exons, introns and 3′ UTRs that contain 1 Alu, >1 Alu in the same orientation, and IR Alu. **(C)** The lariat levels of introns with IR Alu in WT and *DBR1* KO samples. P value was from *t* test. **P < 0.01.

We sought to better understand the divergence between observed and expected structure that human introns exhibited in Fig. 1 E. The proliferation of short interspersed nuclear elements, such as Alu, within introns could be a major contributing factor to the formation of intronic secondary structure. The presence of multiple Alu elements inserted within a single intron in an inverted orientation creates the potential for long stretches of base pairing due to their complementarity. To assess the relationship between Alu elements and intron structure, we subdivided the data into the following categories of introns containing: no Alu (152,194 introns), 1 Alu (44,285), multiple Alu in the same insertion orientation (24,687), or multiple Alu with at least one pair in opposite insertion orientations (IR; 87,437). We hypothesized that introns with IR Alu elements would have the largest differences between observed and shuffled MFE, as the opposing orientation of Alu insertions in these introns provides extensive regions of near perfect complementarity. For all intron Alu content categories except IR Alu, there was a very strong correlation between the MFE of the observed intron and sequence-shuffled introns (Fig. 2 A). In contrast, introns with IR Alu had a markedly weaker correlation, with many introns in this category possessing much more extensive structure than the expectation based on the ensemble of shuffled intron sequences. The ability of IR Alu to form long RNA hairpins within introns suggests the large fraction (28%) of introns that contain IR Alu would trigger cytoplasmic dsRNA surveillance pathways if they were exported to the cytoplasm. Quantifying the presence of Alu elements within different gene regions shows that the parts of mRNA transcripts that are exported to the cytoplasm contain fewer Alu than nuclear introns (Fig. 2 B). While the majority of introns contain Alu, ∼10% of 3′ UTR also contain at least one Alu insertion (Fig. 2 B). Lariat mapping of total RNA sequencing (RNA-seq) samples from DBR1 KO and WT cell lines indicate lariat levels for introns containing IR Alu increase 43-fold in the absence of DBR1 activity (Fig. 2 C).

### Lariats accumulate in the cytoplasm in the absence of DBR1

**Figure 3.**
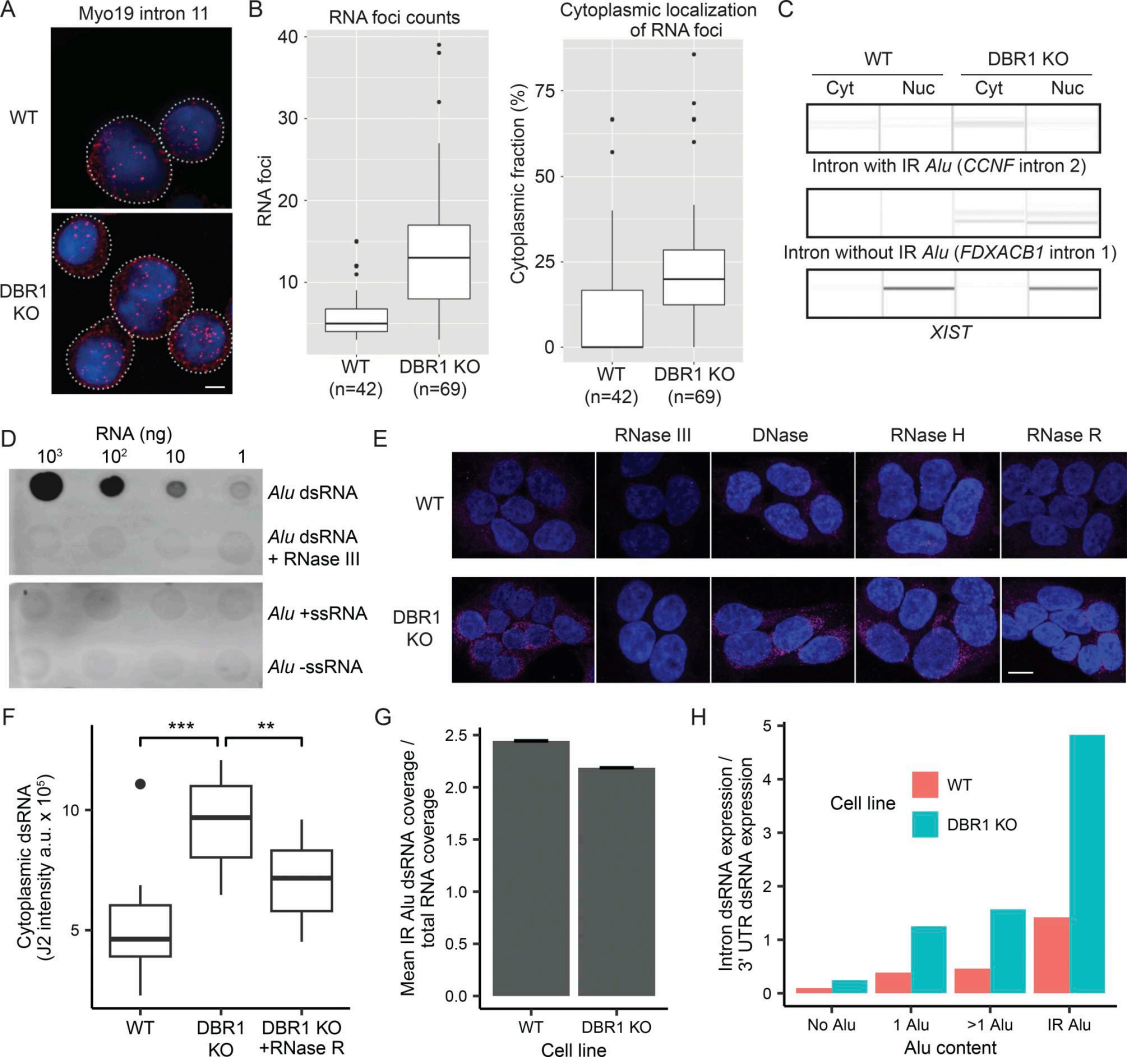
**Lariats accumulate in the cytoplasm and form dsRNA in the absence of DBR1. (A)** Imaging of FISH targeted to *MYO19* intron 11 (red) in WT and DBR1 KO cells. DAPI (blue) served as a marker for nuclei. The white dotted line outlines the cell boundary. The scale bar represents 5 µm. **(B)** Analysis of visualized RNA foci reveals an increase in foci in DBR1 KO samples, as well as a shift to the cytoplasm. *n* represents the number of single cells from at least three independent replicates. **(C)** Lariat PCR reveals specific cytoplasmic enrichment of IR Alu–containing intron lariats in DBR1 KO cells. Nuclear RNA (*XIST*) is used to demonstrate efficient nuclear/cytoplasmic fractionation. Data are representative of three independent experiments. **(D)** Dot blot demonstrating specificity of J2 antibody for Alu dsRNA relative to RNase III–digested Alu dsRNA and Alu ssRNA. **(E)** J2 IF of WT (top row) and DBR1 KO cells (bottom row). Cells were stained for dsRNA (J2, red), with DAPI (blue) serving as a nuclear marker. Representative images of untreated and DNase/RNase-treated cells from at least three biological replicates are shown. The scale bar represents 10 µm. **(F)** Quantification of cytoplasmic dsRNA signal intensity from the IF images in E (P value from *t* test, **P < 0.01, ***P < 0.001). **(G)** Mean ratio of read coverage across IR Alu in cytoplasmic total vs. J2-IP RNA-seq samples. **(H)** For gene regions stratified by the indicated Alu content categories, the relative expression of introns compared with 3′ UTRs in cytoplasmic J2-IP RNA-seq samples. Source data are available for this figure: SourceData F3.

Given that IR Alu sequences can form dsRNA structures, the 43-fold increase in IR Alu-containing lariats suggests potential interactions with cellular dsRNA-sensing mechanisms. As dsRNA sensors, such as MDA5, RIG-I, PKR, TLR3, and OASes, are typically situated in the cytosol (Brubaker et al., 2015; Chen and Hur, 2022; Robinson and Moehle, 2014), a key question is whether lariats that avoid debranching accumulate in the cytoplasm. To measure the distribution of lariats across different subcellular compartments, *MYO19* intron 11 was chosen as an exemplar intron, and its location was visualized through single-molecule fluorescence in situ hybridization (FISH) performed in wildtype and *DBR1* KO cells (Fig. 3 A). Consistent with the elevated level of lariats in the absence of DBR1, the median FISH signal was ∼2.5-fold greater in the *DBR1* KO cells compared with WT cells (Fig. 3 B, left). The cytoplasmic proportion of the FISH signal also increased, suggesting that lariats accumulate in the cytoplasm following a loss of DBR1 activity (Fig. 3 B, right). To directly test whether lariats from IR Alu–containing introns accumulate in the cytoplasm upon DBR1 loss, we performed lariat PCR on fractionated nuclear and cytoplasmic RNA. We observed that lariat derived from an IR Alu–containing intron was specifically enriched in the cytoplasm of *DBR1* KO cells, whereas lariat from a non–IR Alu intron was detected in both the nuclear and cytoplasmic fractions (Fig. 3 C). Furthermore, to investigate whether these lariats can form dsRNA in the cytoplasm, we performed immunofluorescence (IF) staining using the J2 antibody. To ensure J2 specificity for RNA duplexes, we synthesized Alu dsRNA by in vitro transcription (IVT) and confirmed by dot blot that J2 specifically detected the duplex form, but not Alu sense single-stranded RNA (Alu +ssRNA) or Alu antisense ssRNA (Alu –ssRNA) (Fig. 3 D). Moreover, RNase III treatment, which selectively degrades dsRNA, abolished the J2 signal. Having validated antibody specificity, we next performed IF and observed that the cytoplasmic dsRNA signal was increased by ∼1.85-fold in *DBR1* KO cells compared with WT controls (Fig. 3, E and F). After RNase R treatment, which degrades linear RNAs, the cytoplasmic dsRNA signal in DBR1 KO cells was reduced by ∼26%. The retention of the majority (∼74%) of dsRNA signal in these cells is consistent with the presence of some circular RNAs or lariat introns (Fig. 3, E and F). DNase and RNase H treatments did not appreciably alter the intensity of the J2 signal. While these experiments identify dsRNA in the cytoplasm, it is always possible incomplete digestion affects interpretation of these results. Furthermore, J2 identifies IR Alu in vitro, but the signal observed in vivo may arise from unrelated dsRNA. To address this specificity, RNA immunoprecipitation followed by sequencing (RIP-seq) was performed with the J2 antibody on cytoplasmic RNA extracts. Quantification of the RNA-seq alignment coverage at IR Alu insertion sites indicate a 2–2.5× enrichment in the J2-immunoprecipitation (J2-IP) cytosolic samples relative to cytosolic RNA for both cell lines, confirming the antibody’s recognition of endogenous IR Alu (Fig. 3 G). Read coverage of introns and 3′ UTRs in the J2-IP samples was used to estimate the relative dsRNA expression of these regions. In WT cells, the total dsRNA signal originating from IR Alu introns is only slightly greater than the signal from IR Alu 3′ UTRs (Fig. 3 H). In DBR1 KO cells, IR Alu introns have dsRNA expression ∼5 times that of IR Alu 3′ UTRs, indicating the importance of DBR1 for controlling the cytoplasmic accumulation of this dsRNA source.

### DBR1 deficiency attenuates dsRNA sensors

**Figure 4.**
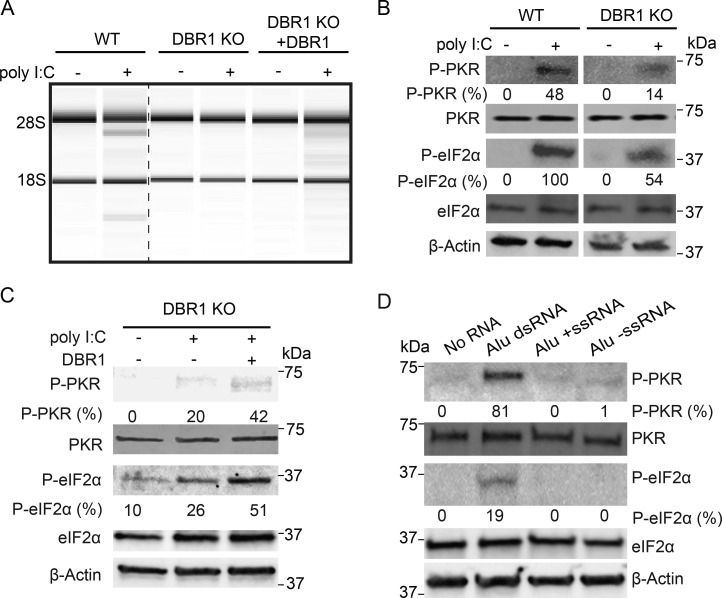
**DBR1 loss suppresses, while Alu dsRNA activates, innate dsRNA-sensing pathways. (A)** Total RNA profiling using an Agilent Bioanalyzer. The absence of rRNA degradation was observed in DBR1 KO cells following 4 h of poly I:C treatment, whereas rRNA degradation was restored upon add-back of DBR1 (DBR1 KO +DBR1) in the presence of poly I:C. Data are representative of three independent experiments. **(B)** In DBR1 KO cells, the phosphorylation levels of PKR and eIF2α were significantly decreased compared with those in WT cells (HEK293T) following 2 h of poly I:C treatment, as determined by western blot analysis. Data are representative of three independent experiments. **(C)** The phosphorylation levels of PKR and eIF2α significantly increased upon add-back of DBR1 following a 1-h treatment with poly I:C, as determined by western blot analysis. Quantification of phosphorylated PKR and eIF2α was performed using ImageJ. Data are representative of three independent experiments. **(D)** Western blot results showed that the phosphorylation of PKR and eIF2α occur specifically in response to Alu dsRNA. Data are representative of three independent experiments. Source data are available for this figure: SourceData F4.

The accumulation of dsRNA from cytoplasmic lariats in DBR1 KO cells results in high levels of cytoplasmic dsRNA, which is a potent stimulator of cellular dsRNA-sensing mechanisms. However, the patient phenotype for DBR1 loss of function includes a hypersusceptibility to viral infection. To determine whether our *DBR1* KO cells recapitulate this phenotype, we transfected WT and *DBR1* KO cells with polyinosinic:polycytidylic acid (poly I:C), a widely used mimic of the pathogenic dsRNAs present during viral infection. We observed greatly reduced rRNA degradation and decreased phosphorylation levels of PKR and eIF2α in *DBR1* KO cells compared with WT cells in response to poly I:C (Fig. 4, A and B). Furthermore, upon reintroducing DBR1 by transfecting KO cells with a DBR1 vector, rRNA degradation activity and PKR and eIF2α phosphorylation levels upon treatment with poly I:C were both restored (Fig. 4, A and C). These results suggest that the elevation of lariats in DBR1-null cells attenuates dsRNA-sensing pathways.

### Alu dsRNA can activate PKR sensor

**Figure S3.**
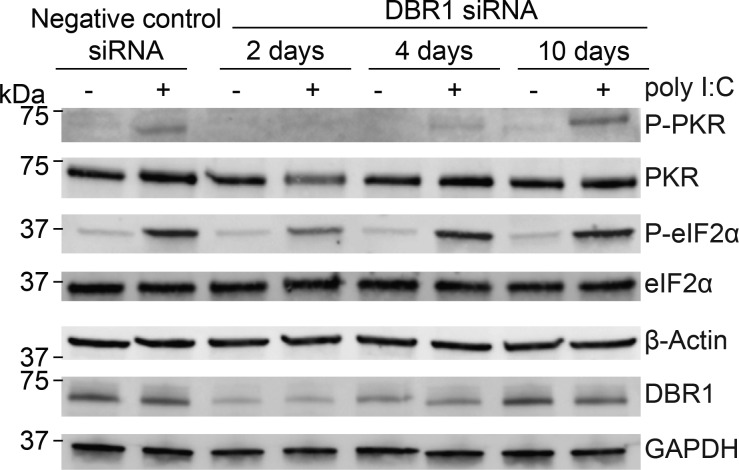
**Time course depletion of DBR1 reveals that PKR activation correlates with DBR1 protein levels.** Western blot analysis showing DBR1 protein levels following siRNA-mediated knockdown over a 10-day time course. Corresponding levels of total and phosphorylated PKR and eIF2α (P-PKR and P-eIF2α) were examined at the indicated time points. Data are representative of three independent experiments. Source data are available for this figure: SourceData FS3.

IR Alus are the most abundant class of cellular dsRNAs (Kim et al., 2018) and can act as immunogenic endogenous dsRNAs (Ahmad et al., 2018; Chung et al., 2018; Wu et al., 2018). To further characterize the role of IR Alus in innate immunity, we transfected IVT Alu dsRNA, validated by J2, into WT cells. The western blot analysis revealed that only Alu dsRNA significantly led to increased phosphorylation of both PKR and eIF2α (Fig. 4 D). In the absence of DBR1, IR Alus embedded in lariats accumulate in the cytoplasm (Fig. 3). Here, we demonstrate Alu dsRNA triggers multiple dsRNA-sensing pathways. Taken together, these two observations would predict chronic stimulation of dsRNA receptors in the absence of DBR1. However, the opposite is true: we find dsRNA sensors that are less responsive in DBR1-deficient cells. Initially, we thought this attenuation was a receptor desensitization from chronic exposure to lariat dsRNA. However, we performed a siRNA time course knockdown of DBR1 (Fig. S3) and found PKR response correlated with the levels of DBR1 protein without the time lag predicted by a model of receptor desensitization.

### High concentrations of Alu dsRNA attenuate PKR activation by titrating PKR dimerization

**Figure 5.**
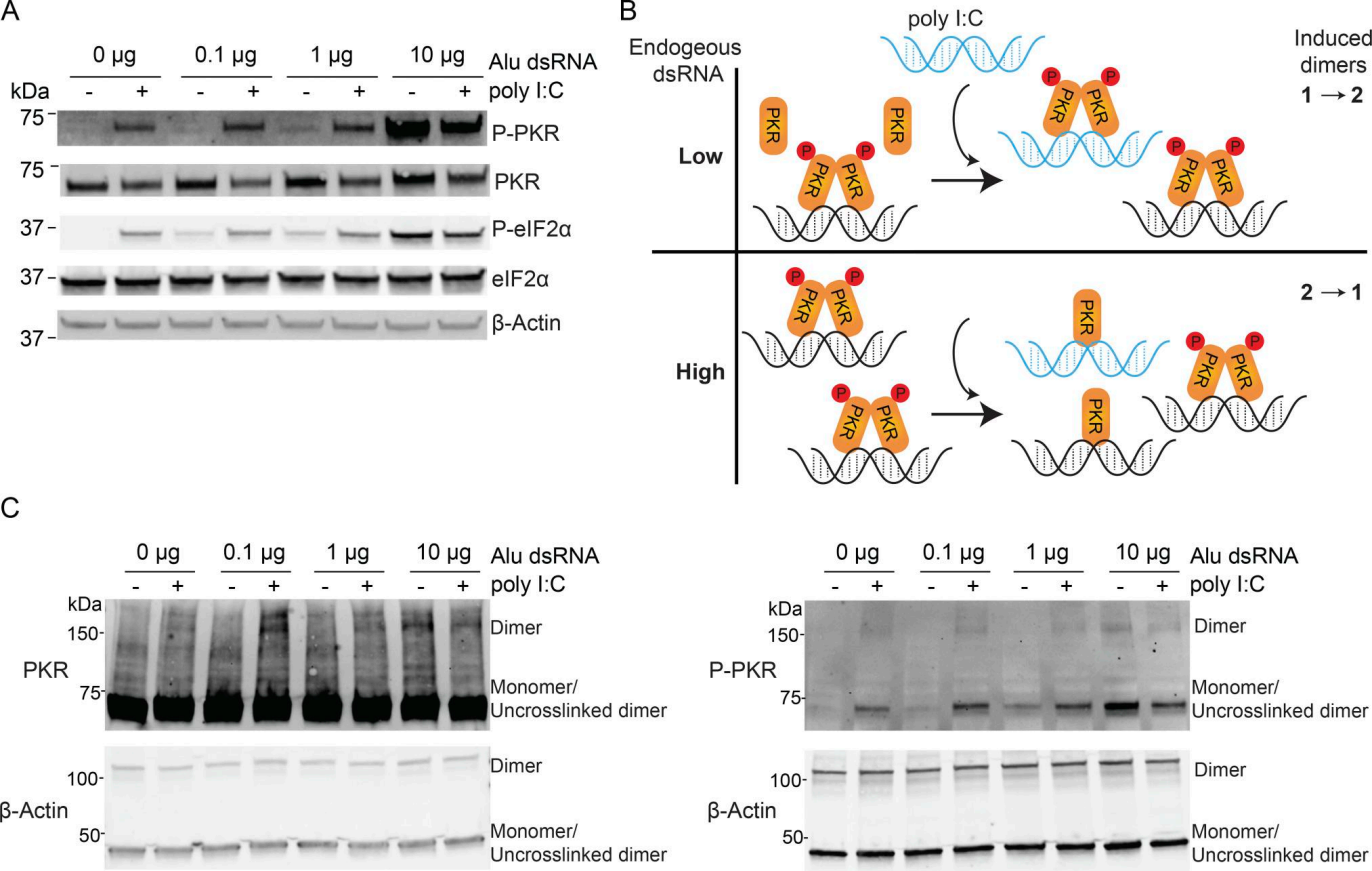
**High levels of Alu dsRNA attenuate PKR activation by titrating PKR dimerization. (A)** Western blot analysis of PKR and eIF2α phosphorylation with varying levels of Alu dsRNA following poly I:C rechallenge. Data are representative of three independent experiments. **(B)** Schematic illustration of the PKR titration model. **(C)** Cells with different levels of Alu dsRNA after poly(I:C) rechallenge were cross-linked with DSS, followed by SDS-PAGE and immunoblotting with anti-PKR (left) and anti–P-PKR (right) antibodies. Data are representative of two independent experiments. Source data are available for this figure: SourceData F5.

**Figure S4.**
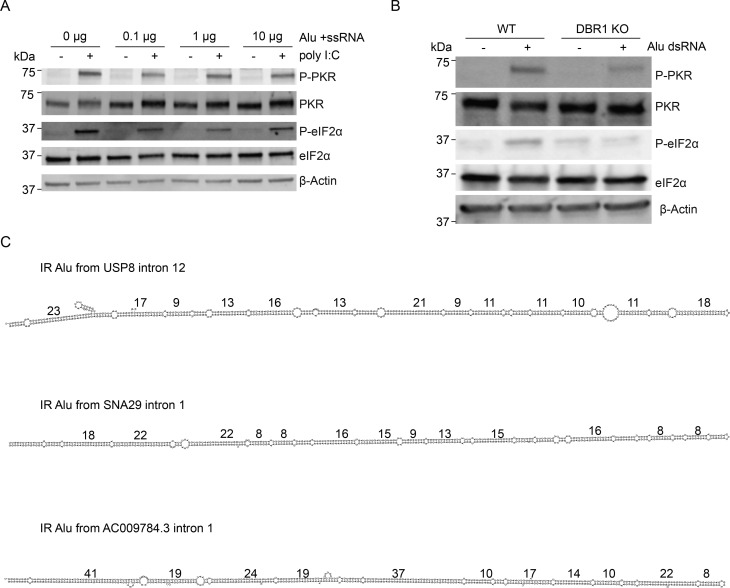
**PKR activation in response to single-stranded and double-stranded Alu RNAs and predicted structures of IR Alus from different introns. (A)** High concentrations of single-stranded Alu RNA do not impair PKR activation. Western blot analysis of PKR and eIF2α phosphorylation with varying levels of Alu ssRNA following poly I:C rechallenge. Data are representative of three independent experiments. **(B)** Reduced PKR and eIF2α phosphorylation in DBR1 KO cells upon Alu dsRNA transfection. WT and DBR1 KO cells were transfected with Alu dsRNA. After 6 h, cell lysates were analyzed by western blot for phosphorylated PKR and eIF2α. Data are representative of three independent experiments. **(C)** Predicted RNA structures of IR Alus from different introns. Numbers indicate the length of the longest continuous duplex region.

To understand the relationship between a cell’s endogenous dsRNA level and ability to respond to exogenous dsRNA, we sought to model the low and high basal levels of cytoplasmic dsAlu seen in DBR1 + and − lines with pre-stimulation Alu dsRNA transfections. WT (HEK293T) cells were transfected with different amounts of Alu dsRNA and, after a 6-h incubation period, were subjected to a second (poly I:C or mock) transfection. The PKR signaling pathway was then tested. Cells treated with low and moderate amounts of Alu dsRNA maintain their ability to react to subsequent transfection with poly I:C, as evidenced by the increased levels of phosphorylated PKR and eIF2α in poly I:C–treated samples (Fig. 5 A). In contrast, transfection with high concentrations of Alu dsRNA interferes with the innate immune response to poly I:C treatment with P-PKR and P-eIF2α levels decreasing in this sample relative to the untreated sample. The control transfection of excessive single-stranded Alu RNA did not produce this attenuation effect (Fig. S4 A). These dynamics, together with the interdimeric phosphorylation mechanism of PKR activation, suggest a titration model of innate immune signaling: When endogenous dsRNA levels are low, unbound immune sensors are present at sufficient levels to respond to external dsRNA stimulus (Fig. 5 B, top), but when the cell has already been subjected to high dsRNA concentrations, exposure to additional dsRNA could redistribute dimerized molecules of PKR across unoccupied dsRNA molecules (which are in excess), thereby preventing productive dimerizations that are required for activation (Fig. 5 B, bottom). Seeking direct evidence of an excess of dsRNA reducing PKR dimers, we performed in vivo disuccinimidyl suberate (DSS) cross-linking followed by western blotting. High level of transfected Alu dsRNA led to a marked accumulation of cross-linked PKR dimers. Upon poly I:C rechallenge, cells with high levels of preexisting Alu dsRNA showed a reduction in PKR dimer signal (Fig. 5 C), indicating that additional dsRNA did not promote further productive PKR–PKR interactions and instead reduced dimers. This pattern aligns with the proposed titration model. Moreover, our DBR1 KO cells, which already harbor high levels of endogenous Alu dsRNA, were transfected with additional Alu dsRNA alongside WT cells. As expected, DBR1 KO cells exhibited lower levels of phosphorylated PKR and eIF2α compared with WT cells, consistent with the titration model (Fig. S4 B).

### Sequence variations in IR Alu decrease the length of dsRNA stretches and reduce PKR activation

**Figure 6.**
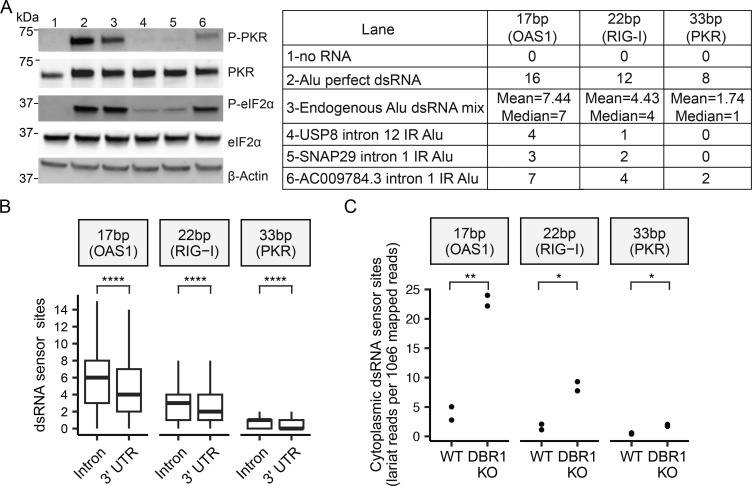
**Sequence variations in IR Alu decrease the length of dsRNA stretches and reduce PKR activation. (A)** Left: Western blot analysis of PKR and eIF2α phosphorylation induced by single (or multiple) IR Alus with different degrees of sequence variation. Data are representative of three independent experiments. Right: Count of dsRNA segments in the predicted MFE structure capable of activating OAS1, RIGI-I, or PKR. **(B)** For IR Alu–containing introns and 3′ UTRs at most 5000 nt long, the predicted MFE structure was used to quantify the distribution of dsRNA lengths capable of activating OAS1, RIG-I, or PKR. P values were from *t* test. ****P < 0.0001. **(C)** Lariat reads were mapped from cytoplasmic RNA-seq samples taken from WT and DBR1 KO cell lines. Structure prediction was performed with RNAfold for each lariat, and the number of lariat-associated dsRNA sites was tallied and normalized by the total mapped reads. P values were from *t* test. *P < 0.05; **P < 0.01. Source data are available for this figure: SourceData F6.

While these experiments utilized perfectly complimentary Alu dsRNA, genetic drift between two elements of an IR Alu creates mismatches. To investigate the effect of structural irregularities, such as mismatches and bulges within IR-Alus, we synthesized Alu + ssRNA and Alu – ssRNA from three different human introns by IVT, reannealed them, and transfected the resulting RNA into cells. Two of these RNAs, predicted to lack long duplex regions (>33 bp) (Fig. S4 C), failed to activate PKR (Fig. 6 A, lanes 4 and 5). Another RNA, containing two predicted longer duplexes (Fig. S4 C), activated PKR less efficiently than Alu perfect dsRNA (Fig. 6 A, lane 6 vs. lane 2). Similarly, a mixture of endogenous Alu dsRNA—formed by annealing sense and antisense Alu RNA amplified from human genomic DNA—was also less efficient at activating PKR than Alu perfect dsRNA (Fig. 6 A, lane 3 vs. lane 2). To assess the prevalence within introns and 3′ UTRs of dsRNA segments from IR Alu capable of activating innate immune sensors, we performed in silico prediction to obtain the MFE structure for each of IR Alu–containing intron and 3′ UTR with length ≤5,000 nt (length was limited due to the computational expense of folding very long RNA sequences). Compared with 3′ UTRs, IR Alu within introns have higher numbers of predicted dsRNA segments with sufficient length to activate OAS1 (17 bp), RIGI-I (22 bp), and PKR (33 bp) despite there being no significant difference in the overall lengths of the two sets of tested regions (Fig. 6 B). This result implies a greater selection against long dsRNA created by IR Alu inserts in 3′ UTR relative to intronic IR Alus. Additionally, normalized counts of dsRNA segments meeting OAS1, RIG-I, and PKR thresholds showed that cytoplasmic lariats from DBR1 KO cells contain more dsRNA sensor sites than those from WT cells (Fig. 6 C).

## Discussion

We previously reported that loss-of-function mutations in *DBR1* impair antiviral responses to HSV-1 infections (Zhang et al., 2018). In that original report, it was noted that lariat abundance increased upon HSV-1 infection regardless of *DBR1* status. Here, we extend this observation to influenza virus, where cellular lariats increase upon influenza infection. It is not clear if the accumulation of host lariats that follows viral infection could act as an immunostimulant. Some DNA methylation inhibitors in chemotherapy appear to bolster an immune response by derepressing endogenous retroviruses (Chiappinelli et al., 2015; Mehdipour et al., 2020). The importance of dsRNA in cell-intrinsic immunity is well established, and our analysis provides evidence for a selective pressure to avoid secondary structure in viral introns (Fig. 1) and human 3′ UTR (Fig. 6). While the GC content of human and viral introns are similar, viral introns are much less structured (Fig. 1, A and B). Viral introns are encoded by an unbalanced composition of nucleotides, such that at least 12% of bases are unpaired in the median viral intron (Fig. 1 C). In contrast, the median human intron has at least 5% of its bases unpaired. It is therefore much less likely for viral introns to fold in a way that achieves the long stretches of dsRNA required to trigger the dsRNA responses in the cytosol. While this difference appears modest, we used these MBP values in simulations to determine the likelihood that a random 17mer (OAS1), 22mer (RIG-I), or 33mer (PKR) had all bases paired. This analysis revealed that viral introns contain these stretches of dsRNA capable of triggering host sensors at 53.7% (OAS1), 50.1% (RIG-I), and 46.1% (PKR) of the frequencies predicted in human introns (Table S1). Some of this difference may be explained by the shorter length of viral introns, but the same trend is observed in our MFE analysis, which is length corrected (Fig. 1 B). Human-intronic sequences folded with more secondary structure than expected by chance, much of it due to the hairpins formed by IR Alu that were long enough to trigger the dsRNA sensors. These hairpins, found in 28% of human introns, are formed from multiple Alus inserted in opposite orientations within the same intron (Fig. 2). While these IR Alus have been recognized as the major source of endogenous dsRNA, prior studies mostly focused on these elements in 3′ UTR (Liddicoat et al., 2015). The vast majority of transcribed IR Alus are intronic, but as introns are rapidly processed, they were not thought to be a major source of endogenous dsRNA. Here, we show this may not be correct. Even in WT cells, introns containing IR Alu are more abundant than IR Alu 3′ UTRs in the cytoplasmic dsRNA fraction (Fig.3 H, WT >1) and have more dsRNA sensor-binding sites (Fig. 6 B). However, in the absence of DBR1, there is a 43-fold increase in the abundance of lariats containing these endogenous dsRNA that could be recognized by the dsRNA sensors. For this recognition, lariats need to accumulate in the cytoplasm (confirmed in Fig. 3, A–C) and contribute to increased cytoplasmic dsRNA signal (confirmed in Fig. 3, E and F). In the absence of DBR1, the lariats from these introns can escape to the cytosol and act as an endogenous source of dsRNA that interacts with cellular pathogenic dsRNA sensing. Performing RIP-seq with the J2 dsRNA antibody on the cytosolic fraction of RNA results in enrichment of Alu dsRNAs (Fig. 3 G). Interestingly, dsRNA that originates from introns with IR Alus is slightly more abundant than the dsRNA from their 3′ UTR counterparts in WT cells. After *DBR1* KO, the dsRNA expression of IR Alu introns increases to five times the level of IR Alu 3′ UTR (Fig. 3 H). This increase in dsRNA has also been seen with small molecular inhibition of the spliceosome and knockdown of splicing factors that presumably expose dsRNA sensors to intron duplexes comprised of IR Alus (Bowling et al., 2021; Zheng et al., 2024). We observe similar effects across an array of different innate immune sensors. DBR1 KO cells exhibited impairments in RNase L and PKR responses to dsRNA (Fig. 4).

Cellular accumulation of dsRNA is a common molecular signature associated with viral infections (i.e., pathogen-associated molecular pattern), and its presence in the cell typically activates dsRNA sensors like MDA5, RIG-I, and PKR, leading to an antiviral response. However, the sensitivity of the dsRNA sensor needs to be sufficiently low to prevent false activation by endogenous sources of dsRNA. To add to the complexity, dsRNA recognition plays a biological role during mitosis—PKR activation shuts down translation during M phase (Kim et al., 2014; Kim et al., 2018). We demonstrated that double-stranded Alus are potent ligands for recognition by PKR sensor (Fig. 4 D). However, in our study, we found that the excessive accumulation of these ligands (i.e., structured IR Alu–containing lariats) in the absence of DBR1 attenuates dsRNA sensors like RNase L and PKR. Our observations that cells with accumulated IR Alu lariats show reduced PKR phosphorylation upon poly I:C treatment support a PKR titration model. In this model, when cells are already exposed to high concentrations of dsRNA, additional dsRNA can lead to a redistribution of PKR binding across separate dsRNA molecules (Fig. 5 B). We demonstrate a loss of poly I:C response in cells pretreated with excessive IR Alu dsRNA (Fig. 5 A). Furthermore, the loss of PKR dimers and activated dimers (Fig. 5 C) suggests the inhibitory effect of excess dsRNA is acting at the step of PKR dimer formation on dsRNA.

**Figure S5.**
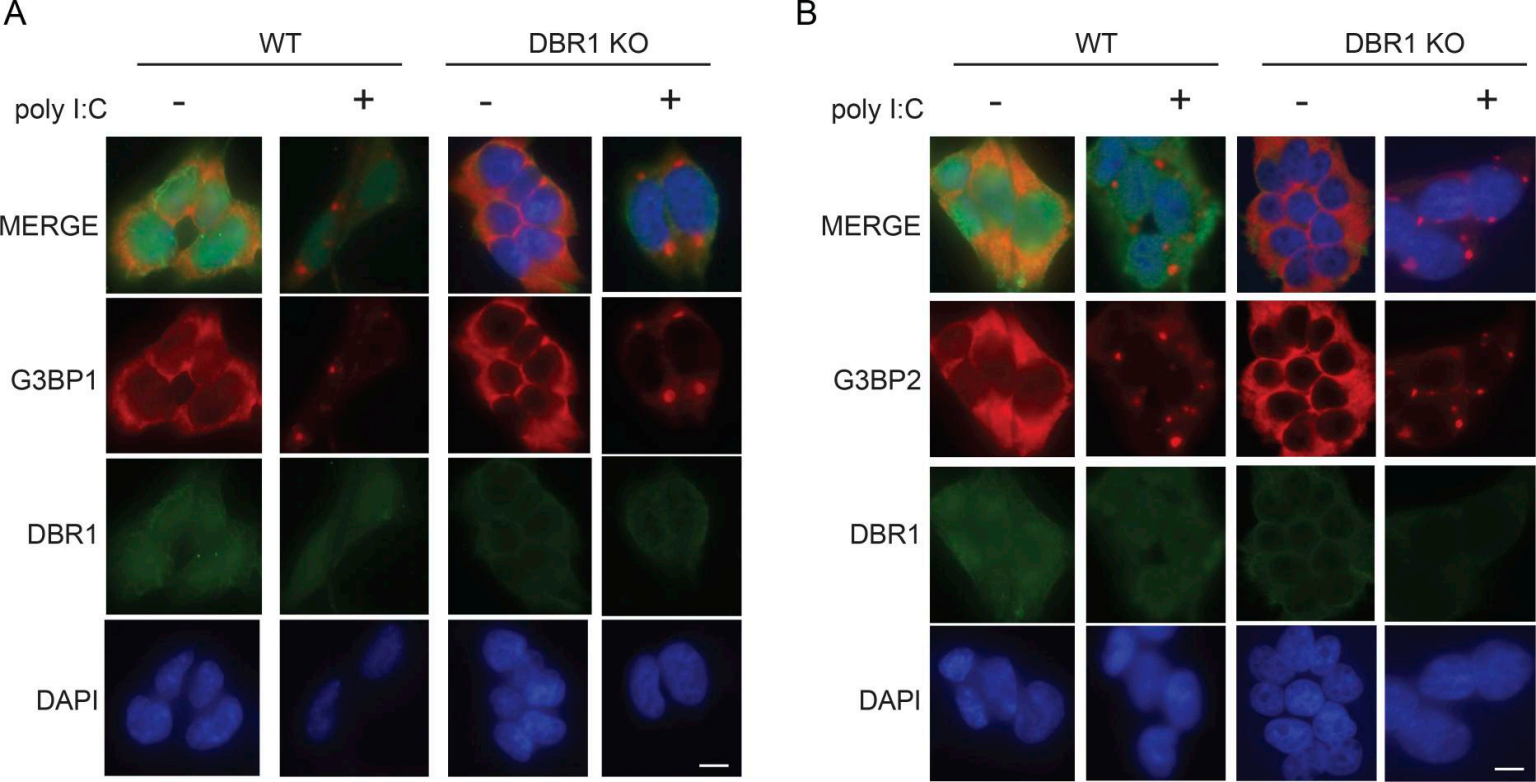
**Stress granule formation in WT and DBR1 KO cells.** **(A and B)** IF staining of G3BP1 (A) and G3BP2 (B) in WT and DBR1 KO cells. Data are representative of at least three independent experiments. The scale bar represents 10 µm.

As IR Alu insertion sites undergo genetic drift, most IR Alus do not form perfect helices. We demonstrate how more realistic versions of Alu hairpins are less activating than perfect hairpins and that there is substantial variation in activation efficiency between IR Alu pairs (Fig. 6 A). Finally, the comparison of 3′ UTR IR Alu with intronic IR Alu shows shorter dsRNA segments with fewer predicted dsRNA sensor-binding sites in 3′ UTR IR Alus that are presumably adapted for the cytoplasm (Fig. 6 B). As 3′ UTR in mRNA spend most of their life cycle in the cytoplasm, this difference suggests purifying selection against dsRNA in the cytoplasm, similar to the results from prior studies on longer repeats (Barak et al., 2020). While both studies show a greater degree of selection on IR Alu in 3′ UTR, Barak et al. still finds selection against intronic structures. Our finding that in WT cells introns with IR Alu are in total a more abundant source of cytoplasmic dsRNA compared with IR Alu 3′ UTRs supports the idea that introns may be under negative selection to avoid structure. The selection on each intron may be small as the large number of IR Alu introns results in each intron comprising a smaller fraction of the pool than each IR Alu 3′ UTR. However, selection on viral introns is easier to detect because of large population size, high mutability, and short generation time. The reduced RNA secondary structure in viral introns may reflect the degree to which this lariat leakage into the cytoplasm can drive selection. Given the role of PKR activation in mitosis, the attenuated PKR we observe in the absence of DBR1 may result in the growth defects observed in DBR1 KO cells (Buerer et al., 2024; Kim et al., 2014; Kim et al., 2018). Consistent with our study, the most recent research demonstrated that RNA lariat accumulation in DBR1-deficient cells hinders PKR activation (Ru et al., 2025). The authors found that accumulated RNA lariats resulting from DBR1 insufficiency impair stress granule assembly and reduce PKR activation by promoting the degradation of G3BP1 and G3BP2, which are crucial for stress granule formation. However, we did not detect reduced stress granule assembly upon poly I:C transfection in our DBR1 KO cells (Fig. S5). We speculate that this may be because PKR inhibition of activation occurs upstream of translation inhibition. Together, these findings suggest that RNA lariat accumulation dampens PKR activation through multiple pathways, highlighting the complex nature of the interactions between RNA lariats and the antiviral response.

## Materials and methods

### Influenza infection

DBR1 I120T/I120T and DBR1 Y17H/Y17H human primary fibroblasts cell lines were from a previous study (Zhang et al., 2018). For influenza infection, 4 × 10^5^ primary fibroblasts per well were added to 6-well plates and infected with influenza, at a multiplicity of infection (MOI) of 5, in DMEM supplemented with 2% fetal calf serum (FCS) (Everett, 1984; Gelman and Silverstein, 1985). After 2 h, the cells were washed and incubated in 500 μl of medium. Cells were collected at 8 h and 24 h and frozen.

### Viral intron analysis

Sequence data for viral introns were obtained via the NCBI Virus database (“RefSeq” > “*Homo sapiens* [human], taxid:9606”) (Hatcher et al., 2017). The locations and sequences were extracted from the GenBank data. This dataset was filtered to remove duplicates, resulting in a final dataset of 127 viral introns. Sequence data for 210,290 human introns from the consensus coding sequence (CCDS) annotation were obtained via the UCSC Table Browser tool (“Feb. 2009 (GRCh37/hg19” > “Genes and Gene Predictions” > “CCDS” > “ccdsGene”) (Karolchik et al., 2004).

MBP can be calculated as follows:

A, T, C, and G = proportions of each nucleotide in sequence

MBP = 1 – [max(A − T, 0) + max(C − G, 0) + |max(T − A, 0) − max(G − C, 0)|]

For example: sequence = 5′-AAAATTCC-3′

A = 0.5, T = 0.25, C = 0.25, G = 0

MBP = 1 – [max(0.5 − 0.25, 0) + max(0.25 – 0, 0) + |max(0.25 – 0.5, 0) – max(0 − 0.25, 0)|]

MBP = 1 – (0.25 + 0.25 + |0 – 0|) = 0.5

For comparisons of observed with shuffled sequences, sequences were randomly shuffled 10 times followed by RNA folding prediction described below.

### RNA secondary structure prediction

Viral introns along with human introns and 3′ UTRs ≤5,000 nt in length were processed with RNAfold from the ViennaRNA package using default options (Lorenz et al., 2011). Length was limited due to the high computational cost of in silico structure prediction for very long RNA sequences. The MFE of each output structure was normalized by the length of the region to obtain comparable MFE values. When computing the count of dsRNA segments within a given region capable of activating innate immune sensors, the following minimum dsRNA lengths were used: OAS1 = 17 bp, RIG-I = 22 bp, and PKR = 33 bp. The predicted structure for each region was split into its component dsRNA stretches, and the total dsRNA sensor-binding sites for a given sensor was calculated as ∑_i floor(length of dsRNA stretch i/minimum activating length of sensor).

### CRISPR DBR1 KO

DBR1 KO cell lines were derived from HEK293T cells as described by Buerer et al. (2024). Cells were cultured in DMEM and 10% FBS on 10-cm plates 24 h prior to transfection at 40% confluency. Each plate was transfected with either 3.6 µg *DBR1* sgRNA (5′-AGA​CGC​TGG​CGC​TGG​CAG​AG-3′) or scramble control (OriGene) as well as 3.6 µg GFP-puro donor DNA (OriGene), 15 µg GeneArt Platinum Cas9 nuclease (Thermo Fisher Scientific), and 43 μl Lipofectamine 2000 (Thermo Fisher Scientific). 48 h after transfection, cells were split 1:10 every 3 days (seven times in total), and single colonies were expanded under 0.5 µg/ml puromycin selection. Minute Total Protein Extraction kit (Invent Biotechnologies) was used, and DBR1 KO was confirmed on single colonies #19 (C19) and #22 (C22) by anti-Dbr1 western blot.

### RNA-seq and lariat identification

Total RNA was extracted with the miRNeasy Mini Kit (Cat# 217004; QIAGEN) and treated with DNase (Ambion) to remove residual genomic DNA. Total RNA quality and integrity were assessed using the Agilent 2100 Bioanalyzer with the RNA Nano kit (Ibberson et al., 2009). RNA-seq libraries were prepared with the Illumina RiboZero TruSeq Stranded Total RNA Library Prep Kit (Cat# RS-122-2201; Illumina) and sequenced on the Illumina NextSeq platform in the 150 nt paired-end configuration. Each library was sequenced three times.

Sequencing reads (GEO accession code GSE195586) were processed using previously described lariat mapping scripts (Buerer et al., 2024). First, reads are filtered out if they contain >5% ambiguous characters. Then, reads are mapped to the genome, and aligned reads are discarded. A mapping index is then created based on the unaligned reads, and a FASTA file containing the first 20 nt of each annotated intron in the transcriptome is mapped to the unaligned reads. Reads are then identified where only one 5′ splice site maps to them, and the alignment has no mismatches or indels. These reads are then trimmed of the sequence from the start of the 5′ SS alignment to the end of the read, and reads where the trimmed portion is <20 nt are filtered out. The remaining trimmed reads are mapped back to the genome, and the trimmed read alignments are then filtered to only consider those with ≤5 mismatches, ≤10% mismatch rate, and no more than one indel of ≤3 nt. For each trimmed read, the highest scoring alignment was chosen after restricting to alignments in the same gene as the 5′ SS alignment and those with the expected inverted mapping order of the 5′ and 3′ segments. The end of this highest scoring alignment is then taken to be the branchpoint of the lariat, from which the read is derived.

### FISH

To visualize lariat RNA species from intron 11 of the *MYO19* gene, Quasar 570–labeled smRNA-FISH probes were ordered from Stellaris RNA-FISH (LGC Biosearch Technologies). smRNA-FISH was performed according to the manufacturer’s instructions. Briefly, 1 × 10^6^ cells were fixed in 3.7% (vol/vol) formaldehyde and then permeabilized in 70% ethanol at 4°C. For in situ hybridization, permeabilized cells were incubated with 1.25 µM probes in the hybridization buffer (10% formamide, 10% dextran sulfate, 1 mg/ml *Escherichia coli* RNA, and 0.2 mg/ml BSA in 2× SSC) at 37°C overnight. Afterward, cells were washed sequentially with wash buffer A (10% formamide in 2× SSC), wash buffer B (0.1% Triton X-100 in 2× SSC), wash buffer C (1× SSC with 1 µg/ml DAPI), and 1× PBS. Finally, these cells were plated onto the coverslip and mounted in ProLong Gold Antifade Mountant (Cat# P36930; Thermo Fisher Scientific). The mounted cells were imaged using an Olympus IX81 inverted widefield microscope equipped with Hamamatsu Orca Flash 4.0 camera with 4 megapixels and a 100 × 1.45 NA oil objective lens. Single RNA molecule counting was done using ImageJ, and statistical analysis was conducted in R.

### IF

For J2 staining, cells were seeded in 24-well and grown on coverslips for 24 h, fixed for 15 min using 4% PFA/PBS. Fixed cells were permeabilized for 15 min using 0.2% Triton X-100/PBS. Cells were treated with 10 U/ml DNase (Cat# M0303S; New England Biolabs), RNase H (Cat# M0297L; New England Biolabs), 20 U/ml RNase III (Cat# M0245L; New England Biolabs), and RNase R (Cat# RNASR-200; MCLAB) for 2 h at 37°C. Cells were then blocked in 5% BSA/PBST for 1 h. Primary antibody incubation was carried out overnight at 4°C using J2 (Cat# 76651L; Cell Signaling Technology) and secondary antibody Donkey anti-Mouse IgG, Alexa Fluor 647 (Cat# A31571; Thermo Fisher Scientific) for 1 h at room temperature.

### Microscopy and image analysis

Cells were imaged using a Zeiss LSM 800 Confocal Microscope. Images of fixed cells were acquired as 32-slice z-stacks with 0.2-µm intervals. Signal intensities were quantified using Fiji software. To calculate the average cytoplasmic signal intensity, the signal from the DAPI-stained nuclei was subtracted from the total signal of the image, and the result was divided by the number of cells in the field.

### In vitro synthesis of different types of Alu dsRNAs

The consensus Alu DNA sequence (5′-GGC​CGG​GCG​CGG​TGG​CTC​ACG​CCT​GTA​ATC​CCA​GCA​CTT​TGG​GAG​GCC​GAG​GCG​GGC​GGA​TCA​CCT​GAG​GTC​AGG​AGT​TCG​AGA​CCA​GCC​TGG​CCA​ACA​TGG​TGA​AAC​CCC​GTC​TCT​ACT​AAA​AAT​ACA​AAA​ATT​AGC​CGG​GCG​TGG​TGG​CGG​GCG​CCT​GTA​ATC​CCA​GCT​ACT​CGG​GAG​GCT​GAG​GCA​GGA​GAA​TCG​CTT​GAA​CCC​GGG​AGG​CGG​AGG​TTG​CAG​TGA​GCC​GAG​ATC​GCG​CCA​CTG​CAC​TCC​AGC​CTG​GGC​GAC​AGA​GCG​AGA​CTC​CGT​CTC-3′) was synthesized by Twist Bioscience. For the preparation of sense and antisense Alu templates, a T7 promoter sequence was appended to the 5′ end of the forward or reverse primer, respectively. PCR products were purified using the QIAquick PCR Purification Kit (Cat# 28106; Qiagen). IVT of Alu +ss and Alu –ss RNAs was performed using the HiScribe T7 Quick High Yield RNA Synthesis Kit (Cat# E2050S; New England Biolabs). The synthesized RNAs were subsequently treated with DNase I to remove residual DNA template, followed by treatment with calf intestinal phosphatase (Cat# M0525S; New England Biolabs) to eliminate 5′-triphosphates. Final products were purified using the Monarch RNA Cleanup Kit (Cat# T2030L; New England Biolabs). To generate Alu perfect dsRNA, equimolar amounts of Alu +ss and Alu –ss RNAs were mixed in annealing buffer (10 mM Tris-HCl, pH 7.5; 100 mM NaCl), denatured at 75°C for 5 min, and slowly cooled to room temperature to allow duplex formation.

Endogenous Alu dsRNA mix and IR Alus from human introns were prepared in the same manner by PCR amplification of their respective templates from human genomic DNA (Cat# G3041; Promega), followed by IVT and annealing of the positive and negative strand RNAs.

### RNA dot blot

The Alu perfect dsRNA, Alu +ssRNA, and Alu −ssRNA were serially diluted and dotted onto a nylon membrane. In parallel, the Alu dsRNA was treated with RNase III (New England Biolabs) according to the manufacturer’s protocol. The RNAs were cross-linked to the membrane using UV light at 125 mJ/cm^2^ at 254 nm. The blot was probed with the dsRNA-specific J2 antibody (Cat# 76651L; Cell Signaling Technology).

### Subcellular fractionation, lariat PCR, and immunoprecipitation of dsRNA

Subcellular fractions were prepared as previously described (Bhatt et al., 2012), with modifications. Briefly, 1 × 10^7^ cells were gently trypsinized, collected from the plate, and pelleted by centrifugation at 1,500 rpm for 2 min at 4°C. Pellets were resuspended in 200 μl cold cytoplasmic lysis buffer (0.15% NP-40, 10 mM Tris, pH 7.5, and 150 mM NaCl) and incubated on ice for 5 min. Lysates were layered onto 500 μl cold sucrose buffer (24% wt/vol sucrose, 10 mM Tris, pH 7.5, and 150 mM NaCl) and centrifuged at 13,000 rpm for 10 min at 4°C. The supernatant was collected as the cytoplasmic fraction.

The nuclear pellet was resuspended in 200 μl cold glycerol buffer (20 mM Tris, pH 8.0, 75 mM NaCl, 0.5 mM EDTA, 50% glycerol, and 0.85 mM DTT), followed by addition of 200 μl cold nuclei lysis buffer (20 mM HEPES, pH 7.6, 7.5 mM MgCl_2_, 0.2 mM EDTA, 0.3 M NaCl, 1 M urea, 1% NP-40, and 1 mM DTT). The mixture was pulse-vortexed, incubated on ice for 2 min, and centrifuged at 14,000 rpm for 2 min at 4°C. The resulting supernatant was collected as the nucleoplasmic fraction.

RNA from both cytoplasmic and nucleoplasmic fractions was isolated and purified using the RNeasy Mini Kit (Cat# 74104; Qiagen) according to the manufacturer’s instructions. Following DNase treatment, cDNA was synthesized using SuperScript IV Reverse Transcriptase (Cat# 18090050; Thermo Fisher Scientific). Lariat PCR was then performed on the cDNA using a nested amplification strategy. The primers for lariat PCR were listed in Table 1.

**Table 1.** Primer sequences used in this study

Primer name	Primer sequence (5′–3′)
FDXACB1 lariat F1	5′-CGT​CCC​AAA​GTG​GAA​AGG​GTG-3′
FDXACB1 lariat R1	5′-CGC​TGG​GCG​GGG​TTT​C-3′
FDXACB1 lariat F2	5′-GCG​TGG​GGA​TGT​AAC​CCA​CG-3′
FDXACB1 lariat R2	5′-AGA​GGG​GCG​GGC​TCG-3′
CCNF lariat F1	5′-GAC​CTC​AAC​TAC​TTG​GGA​TG-3′
CCNF lariat F2	5′-TCA​AGA​GCC​TCC​AGG​T-3′
CCNF lariat R1	5′-AGC​CAA​AGT​TTT​GTT​CTG​AC-3′
CCNF2 lariat R2	5′-CAC​AAG​GCC​TGA​TGG​TAT​AAT-3′
XIST F	5′-GTG​TTA​GTG​ATC​CAT​TCC​CTT​TGA-3′
XIST R	5′-CAG​GCA​TGT​TGA​TCT​TCA​GGT​G-3′

Purified RNA was immunoprecipitated using the dsRNA-specific J2 antibody. Briefly, 5 µg of J2 antibody was incubated with 30 μl of pre-washed Protein A/G magnetic beads (Cat# 88803; Thermo Fisher Scientific) and rotated at 4°C for 1 h to allow antibody bead binding. Then, 5 µg of cytoplasmic RNA was incubated with the antibody bead complex in binding buffer (150 mM NaCl, 50 mM Tris pH 8.0, 1 mM EDTA, and 1% NP-40) and rotated overnight at 4°C. The beads were subsequently washed five times with cold binding buffer, and RNA was extracted using the Monarch RNA Clean up Kit (Cat# T2030L; New England Biolabs).

### RNA-seq of cytoplasmic total and J2-IP RNA

Using HEK293T WT and DBR1 KO cell lines, two replicates each were performed of the cytoplasmic RNA extraction described above. For each replicate, an aliquot of total cytoplasmic RNA was taken, and the remaining cytoplasmic RNA underwent immunoprecipitation with the J2 antibody. The total and J2-IP RNA samples were sent to Azenta Life Sciences for library preparation and RNA-seq. Strand-specific RNA-seq library was prepared using NEBNext Ultra II Directional RNA Library Prep Kit for Illumina following the manufacturer’s instructions. Briefly, the enriched RNAs were fragmented for 8 min at 94°C. First strand and second strand cDNA were subsequently synthesized. The second strand of cDNA was marked by incorporating dUTP during the synthesis. cDNA fragments were adenylated at 3′ ends, and indexed adapter was ligated to cDNA fragments. Limited cycle PCR was used for library enrichment. The sequencing library was validated on the Agilent TapeStation and quantified by using Qubit 4.0 Fluorometer as well as by quantitative PCR. Illumina 2 × 150 bp paired-end sequencing was performed at a target depth of 50 million reads/sample. Adapter trimming and quality filtering were performed using fastp version 0.24.0 with default options (Chen, 2023). Reads were aligned to human genome assembly hg38 with HISAT2 version2.2.1 using option “--rna-strandness RF” (Kim et al., 2019). Reads originating from transcripts on the positive and negative genomic strands were sorted into separate files with samtools version 1.17, and the number of reads aligning to IR Alu insertions within genes on each strand was tallied with bedtools version 2.31.0 (Danecek et al., 2021; Quinlan and Hall, 2010). Similarly, bedtools was used to tally region counts for all introns and 3′ UTR, and these counts were normalized to obtain RPKM values. RPKM values were converted to TPM values, which were used for comparing the relative expression of introns and 3′ UTRs in the J2-IP dsRNA-sequencing samples. The sequencing data have been deposited in the NCBI Sequence Read Archive under accession number PRJNA1400086.

### poly I:C treatment

Both WT (HEK293T) and DBR1 KO cells were cultured in DMEM containing 10% FBS and 100 U/ml P/S. Prior to poly I:C transfection, the culture medium was replaced with serum-free DMEM. Poly I:C (Cat# HY-107202; MedChem Express) was transfected at a final concentration of 1 μg/ml using Lipofectamine 3000 (Invitrogen) in serum-free medium, and cells were incubated at 37°C for the indicated times.

### RNA structure analysis of endogenous Alu mix

Samples of endogenous positive and negative Alu were separately amplified from human genomic DNA (Cat# G3041; Promega) and sent to Massachusetts General Hospital for low-throughput 2 × 142 bp paired-end sequencing. Reads were adapter trimmed and quality filtered using fastp version 0.24.0 with default options (Chen, 2023). Reads were aligned to human genome assembly hg38 with HISAT2 version 2.2.1 using default options (Kim et al., 2019). The read alignments were used to calculate the relative representation of genomic Alus within the positive and negative Alu samples. To determine the distribution of secondary structures assuming random pairing of positive and negative Alus, 10,000 trials of the following procedure were performed: (1) one positive and one negative Alu were sampled with probabilities determined by the relative representation from the read alignments, (2) the hybridization structure of the sampled Alus was computed using RNAduplex from the ViennaRNA package with default options (Lorenz et al., 2011), (3) and the dsRNA stretches from the predicted structure were used to compute the total immune sensor-binding sites of the hybridized Alu pair as described in the Materials and methods section “RNA secondary structure prediction.#x201d;

### Western blot

For DBR1 add-back, 5 μg of DBR1 expression vector (Cat# OHu11162; GenScript) was transfected into cells using Lipofectamine 3000 (Invitrogen) 24 h prior to the poly I:C treatment. Cell lysates were prepared using radioimmunoprecipitation assay lysis buffer containing protease and phosphatase inhibitors. Protein samples were separated on 4–20% Mini-PROTEAN gel (Cat# 4561093EDU and Cat# 4561096EDU; Bio-Rad) and transferred to a polyvinylidene difluoride membrane. The blot was then probed with anti-PKR (phospho T446) rabbit antibody [E120] (Cat# ab32036; Abcam), phospho-eIF2α (Ser51) rabbit antibody (Cat# 9721; Cell Signaling Technology)/anti-EIF2S1 (phospho S51) antibody [E90] (Cat# ab32157; Abcam), anti-PKR antibody [Y117] (Cat# ab32506; Abcam), EIF2S1 recombinant antibody (Cat# 82936-8-RR; Proteintech), and β-actin antibody (Cat# 66009-1-Ig; Proteintech). Goat anti-rabbit IgG (H+L) highly cross-adsorbed secondary antibody, Alexa Fluor Plus 800 (Cat# A32735; Invitrogen) and goat anti-mouse IgG (H+L) highly cross-adsorbed secondary antibody, Alexa Fluor Plus 680 (Cat# A32729; Invitrogen) were used according to the manufacturer’s instructions. The Bio-Rad imager was used to capture the images.

### In vivo cross-linking assay

Cells were washed with ice-cold PBS. DSS (#21655; Thermo Fisher Scientific) was added to a final concentration of 2 mM, and the reaction was incubated for 30 min at room temperature. The cross-linking reaction was then quenched by adding 1 M Tris (pH 7.5) to a final concentration of 20 mM and incubating for 10 min at room temperature. Cell lysates were subsequently analyzed by SDS-PAGE.

### Online supplemental material

Fig. S1 shows cellular lariats increase in DBR1 mutant cells upon influenza virus infection. Fig. S2 shows a comparison of viral and human intron lengths and secondary structure in human transcripts. Fig. S3 shows time course depletion of DBR1 and corresponding changes in PKR activation. Fig. S4 shows PKR activation in response to single-stranded and double-stranded Alu RNAs and predicted structures of IR Alus from different introns. Fig. S5 shows IF staining of G3BP1 and G3BP2 in WT and DBR1 KO cells. Table S1 shows the probability that a random k-mer is fully paired, based on the MBP metric.

## Data availability

All original code has been deposited at GitHub (https://github.com/fairbrother-lab/lariat_virus_paper) and is publicly available as of the date of publication.

## Supplementary Material

Table S1 shows probability that a random k-mer is fully paired based on the MBP metric.

SourceData F3 is the source file for Fig. 3.

SourceData F4 is the source file for Fig. 4.

SourceData F5 is the source file for Fig. 5.

SourceData F6 is the source file for Fig. 6.

SourceData FS3 is the source file for Fig. S3.
