# Supplementary material for: Defects in intron recycling suppress the antiviral response via a mechanism of intronic endogenous dsRNA
Source: J Exp Med. 2026 Mar 12;223(4):e20250344. doi: 10.1084/jem.20250344 (PMC13189227; doi:10.1084/jem.20250344)
Supplement: Table S1 — shows probability that a random k-mer is fully paired based on the MBP metric. [file jem_20250344_tables1.docx]

**Table S1. Probability that a random kmer is fully paired based on the maximum base pairing metric**

|  | **17bp (OAS1)** | **22bp (RIG-I)** | **33bp (PKR)** |
| --- | --- | --- | --- |
| **Human introns** | 43.0% | 36.5% | 27.1% |
| **Viral introns** | 23.1% | 18.3% | 12.5% |

For each human and viral intron, 1000 trials were performed which sampled the pairing of 17-, 22- and 33-mers from the distribution associated with maximal pairing as defined by the maximum base pairing metric (i.e. given intron length L and maximum base pairing m, the pairing status for each base in the kmer was sampled from a list containing L*m ones and L*(1-m) zeroes where a one indicates a paired base). For each intron set, the percentage of sampled kmers which were fully paired is reported.
