# Supplementary material for: Defects in intron recycling suppress the antiviral response via a mechanism of intronic endogenous dsRNA
Source: J Exp Med. 2026 Mar 12;223(4):e20250344. doi: 10.1084/jem.20250344 (PMC13189227; doi:10.1084/jem.20250344)

SourceDataF3C

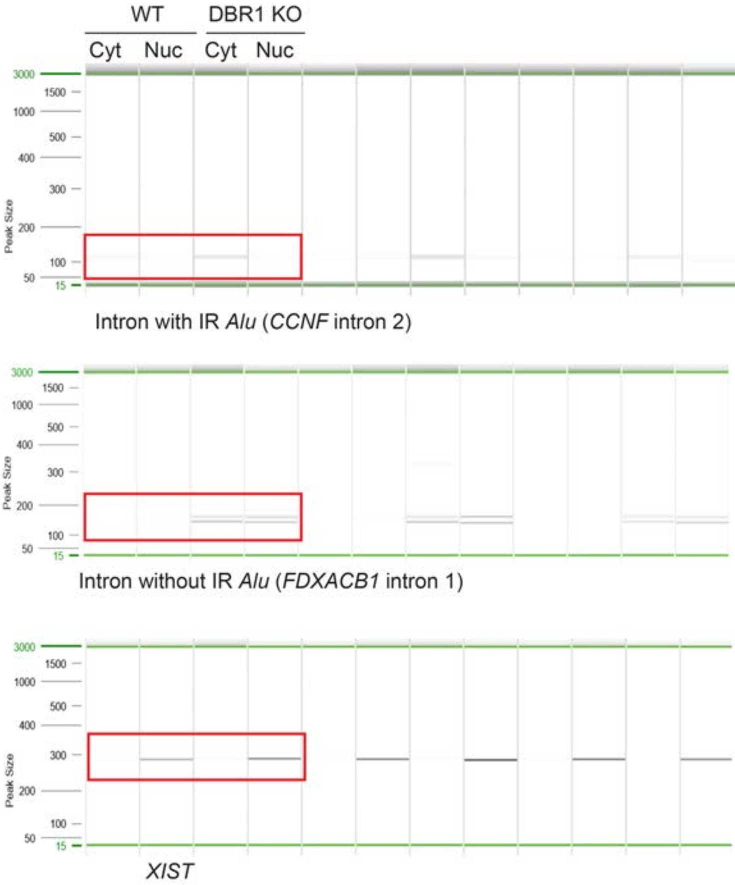

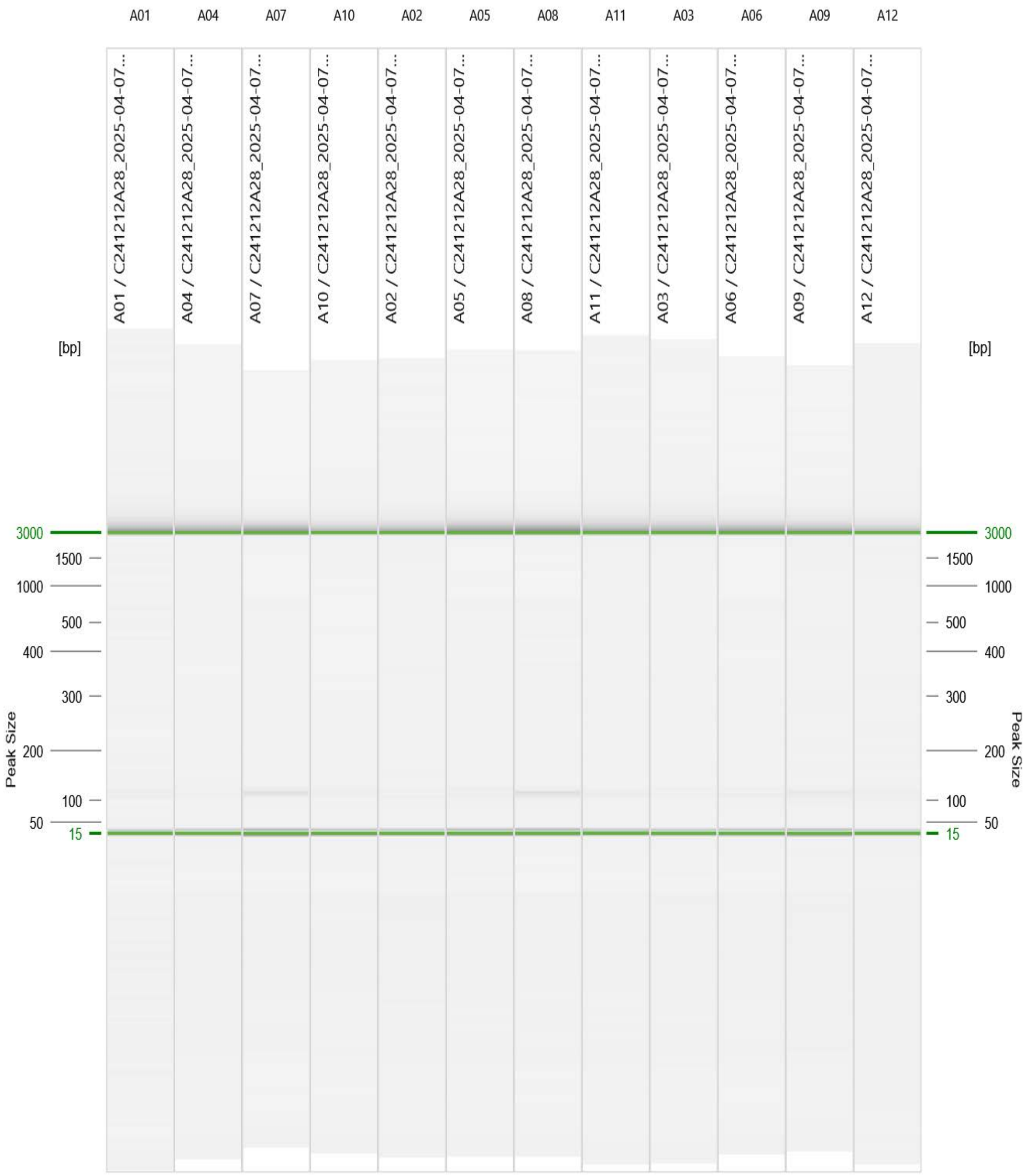
A01
A04
A07
A10
A02
A05
A08
A11
A03
A06
A09
A12
A01 / C241212A28_2025-04-07...
A04 / C241212A28_2025-04-07...
A07 / C241212A28_2025-04-07...
A10 / C241212A28_2025-04-07...
A02 / C241212A28_2025-04-07...
A05 / C241212A28_2025-04-07...
A08 / C241212A28_2025-04-07...
A11 / C241212A28_2025-04-07...
A03 / C241212A28_2025-04-07...
A06 / C241212A28_2025-04-07...
A09 / C241212A28_2025-04-07...
A12 / C241212A28_2025-04-07...
[bp]
3000
1500
1000
500
400
300
200
100
50
15
Peak Size

Figure: 1

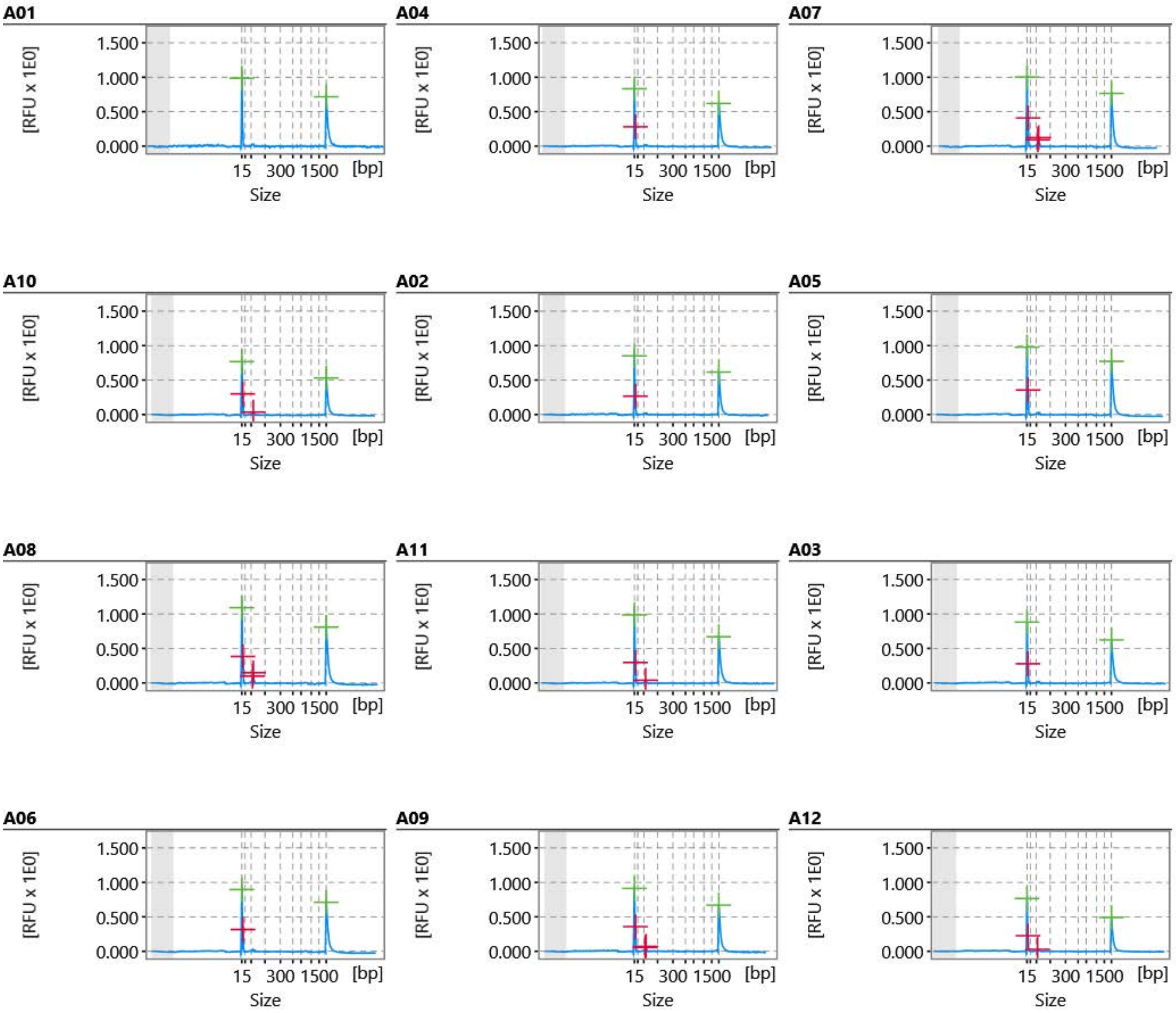
A01
A04
A07
A10
A02
A05
A08
A11
A03
A06
A09
A12
[RFU x 1E0]
1.500
1.000
0.500
0.000
15
300
1500
[bp]
Size

Figure: 3

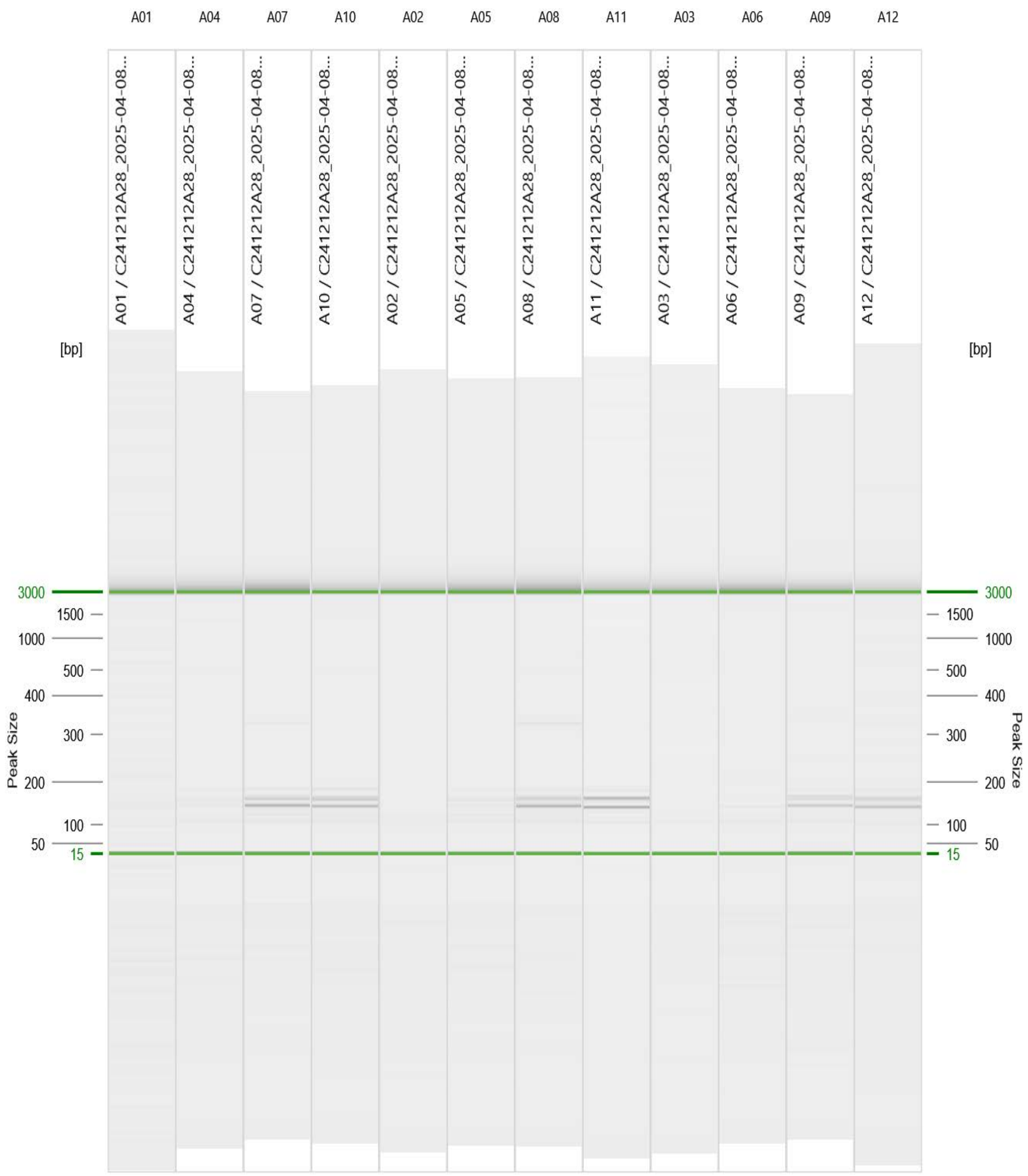
A01 A04 A07 A10 A02 A05 A08 A11 A03 A06 A09 A12
A01 / C241212A28_2025-04-08...
A04 / C241212A28_2025-04-08...
A07 / C241212A28_2025-04-08...
A10 / C241212A28_2025-04-08...
A02 / C241212A28_2025-04-08...
A05 / C241212A28_2025-04-08...
A08 / C241212A28_2025-04-08...
A11 / C241212A28_2025-04-08...
A03 / C241212A28_2025-04-08...
A06 / C241212A28_2025-04-08...
A09 / C241212A28_2025-04-08...
A12 / C241212A28_2025-04-08...
[bp]
Peak Size
3000
1500
1000
500
400
300
200
100
50
15

Figure: 1

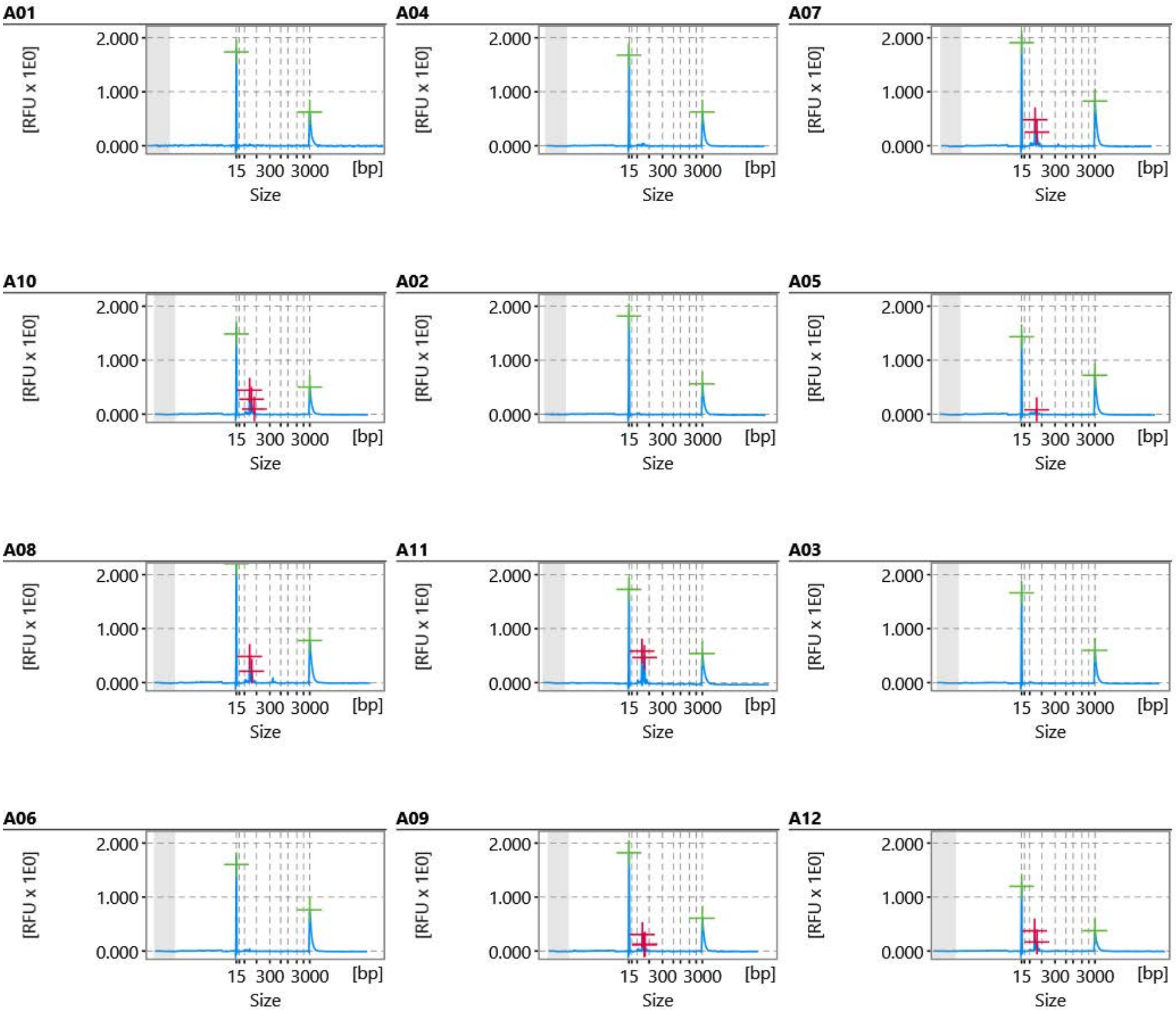

Figure: 2

Overall Smear Result Table											
C241212A28_2025-04-08_13-45-08											
A1		A4		A7		A10		A2		A5	
Median Size	Conc. AoI	Median Size	Conc. AoI	Median Size	Conc. AoI	Median Size	Conc. AoI	Median Size	Conc. AoI	Median Size	Conc. AoI

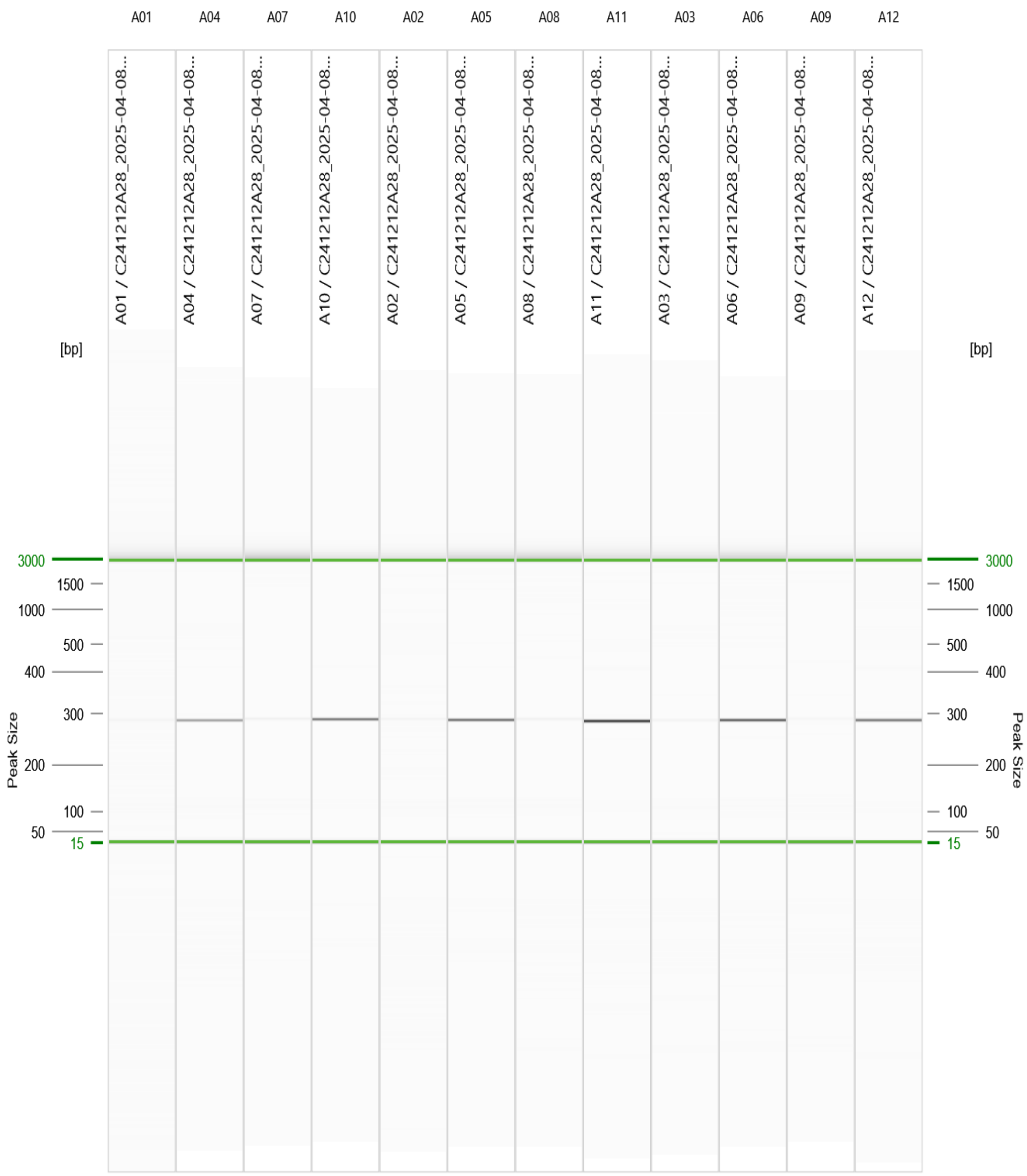
A01
A04
A07
A10
A02
A05
A08
A11
A03
A06
A09
A12
A01 / C241212A28_2025-04-08...
A04 / C241212A28_2025-04-08...
A07 / C241212A28_2025-04-08...
A10 / C241212A28_2025-04-08...
A02 / C241212A28_2025-04-08...
A05 / C241212A28_2025-04-08...
A08 / C241212A28_2025-04-08...
A11 / C241212A28_2025-04-08...
A03 / C241212A28_2025-04-08...
A06 / C241212A28_2025-04-08...
A09 / C241212A28_2025-04-08...
A12 / C241212A28_2025-04-08...
[bp]
3000
1500
1000
500
400
300
200
100
50
15
Peak Size

Figure: 1

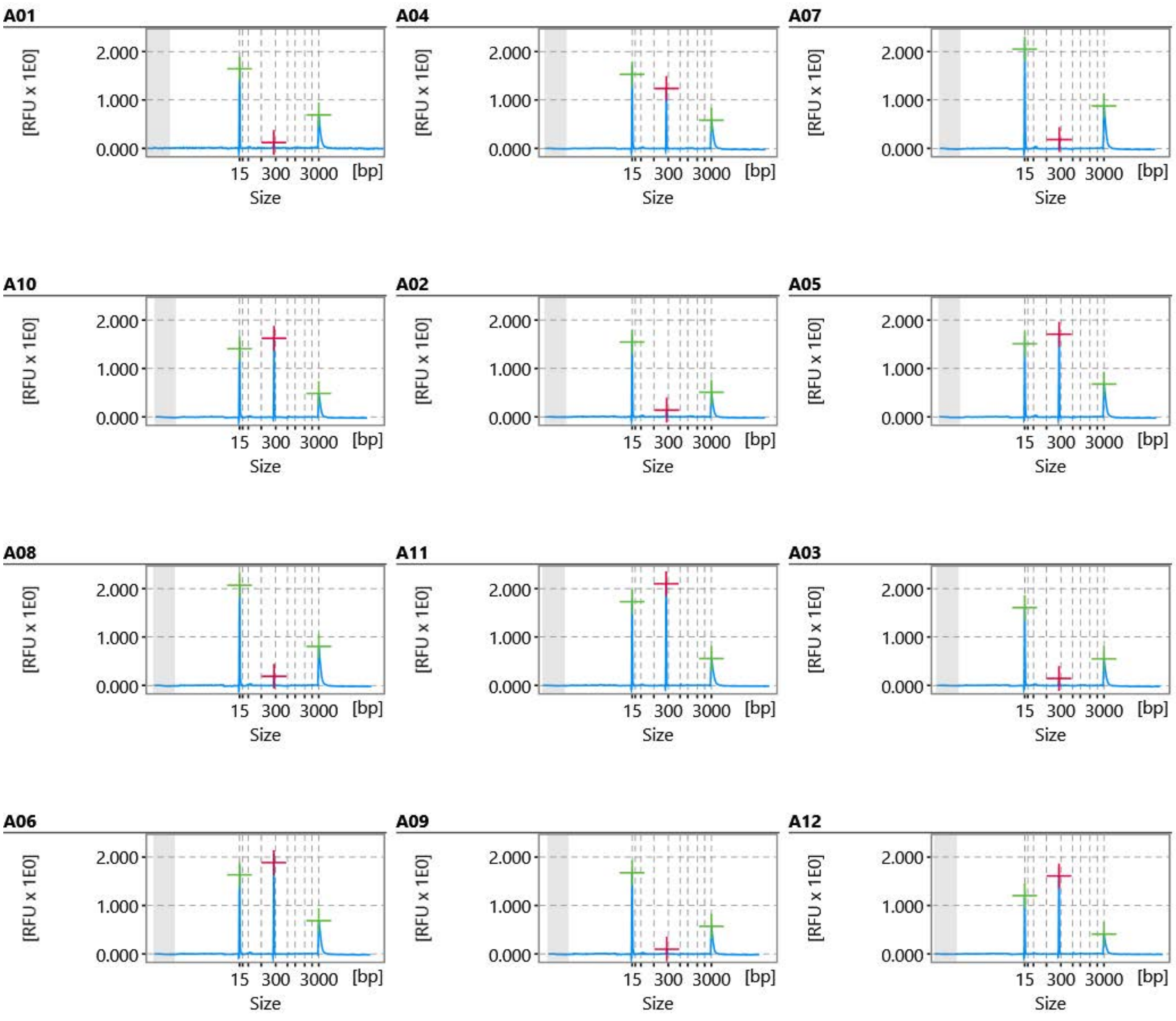
A01
A04
A07
A10
A02
A05
A08
A11
A03
A06
A09
A12
[RFU x 1E0]
2.000
1.000
0.000
15 300 3000 [bp]
Size

Figure: 3

SourceDataF3D

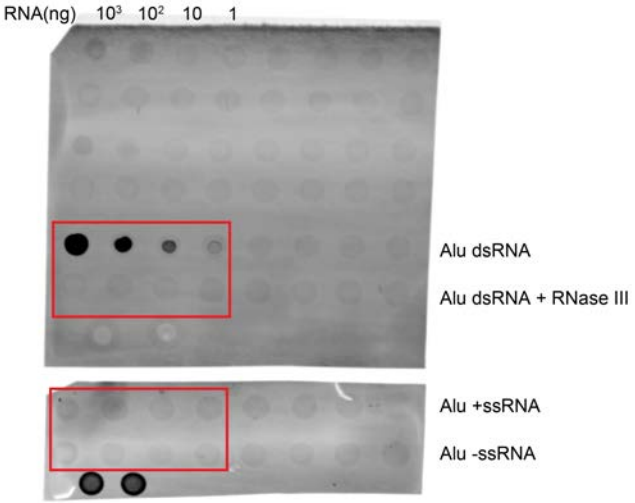

Supplement: SourceData F3 — is the source file for Fig. 3. [file jem_20250344_sourcedataf3.pdf]
