# Supplementary material for: Defects in intron recycling suppress the antiviral response via a mechanism of intronic endogenous dsRNA
Source: J Exp Med. 2026 Mar 12;223(4):e20250344. doi: 10.1084/jem.20250344 (PMC13189227; doi:10.1084/jem.20250344)

SourceDataF4A

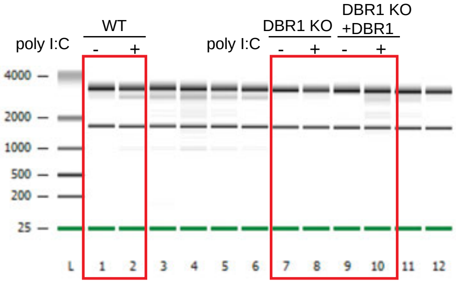

Assay Class: Eukaryote Total RNA Nano
Data Path: C:\...nalyzer\2100 expert\Data\2024-04-08\2024-04-08_13-28-24.xad

Created: 4/8/2024 1:28:23 PM
Modified: 4/8/2024 1:52:15 PM

Gel Image

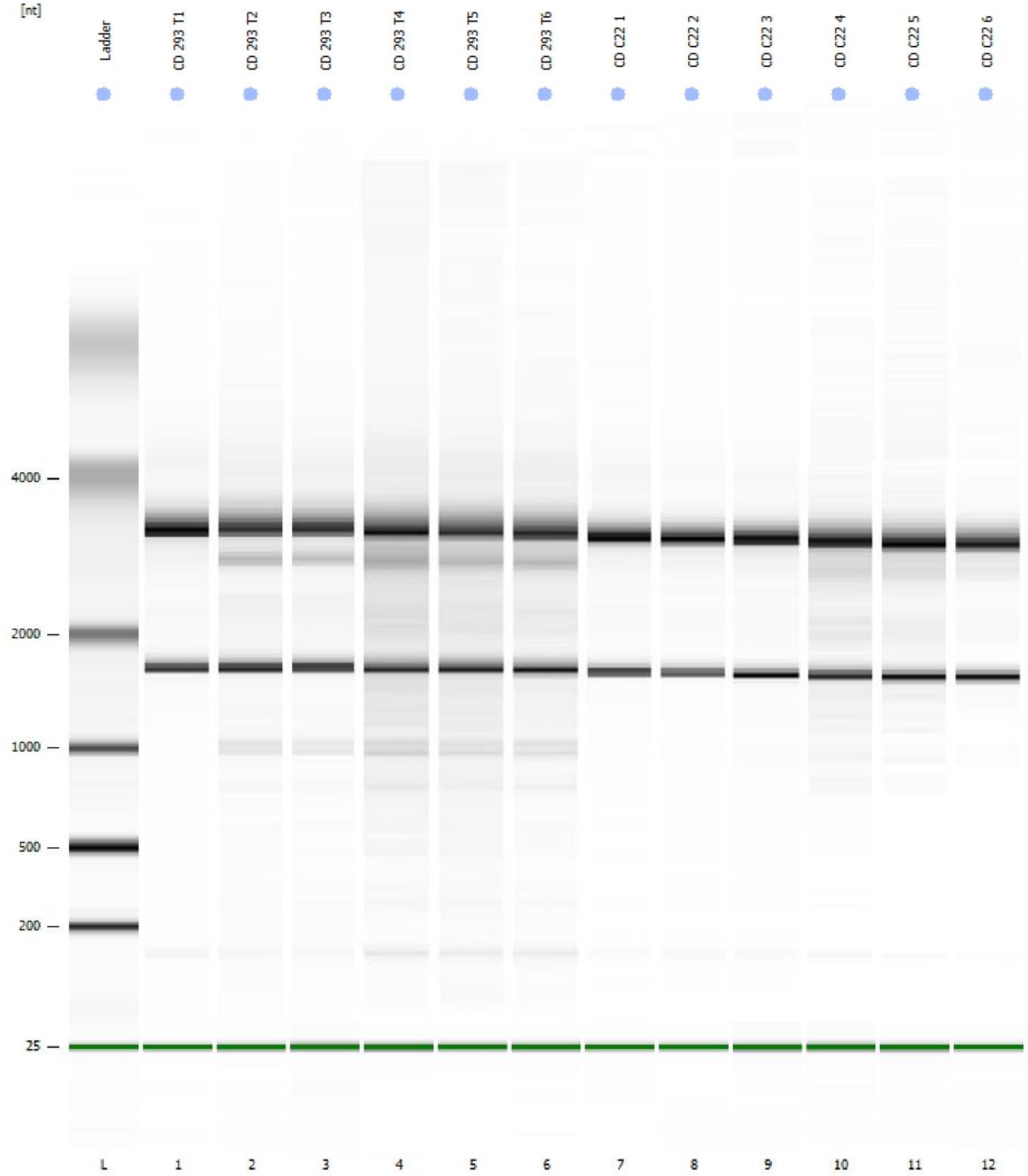

 © Copyright 2003 - 2021 Agilent, Inc.

Assay Class: Eukaryote Total RNA Nano
Data Path: C:\...nalyzer\2100 expert\Data\2024-04-08\2024-04-08_13-28-24.xad

Created: 4/8/2024 1:28:23 PM
Modified: 4/8/2024 1:52:15 PM

Electrophoresis File Run Summary

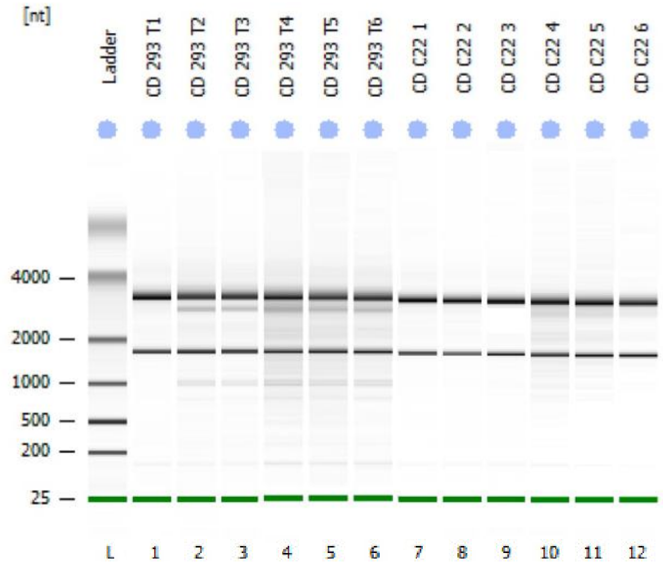

Instrument Information:

Instrument Name: DE72901241 Firmware: C.01.069

Serial#: DE72901241 Type: G2939A

Assay Information:

Assay Origin Path: C:\Program Files (x86)\Agilent\2100 bioanalyzer\2100 expert\assays\RNA\DV200 RNA Nano.xsy

Assay Class: Eukaryote Total RNA Nano

Version: 2.6

Assay Comments: Total RNA Analysis ng sensitivity (Eukaryote)

Modified for DV200 Analysis

Chip Information:

Chip Lot #:

Reagent Kit Lot #:

Chip Comments:

CD 293 T1
RIN:10

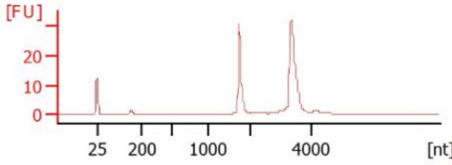

CD 293 T2
RIN: 9.10

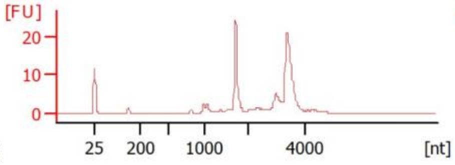

CD 293 T3
RIN: 8.40

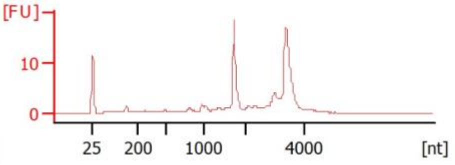

CD 293 T4
RIN: 8.10

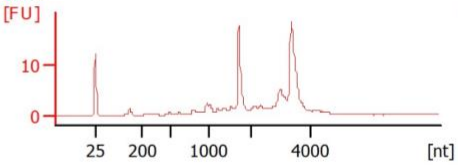

CD 293 T5
RIN: 8.40

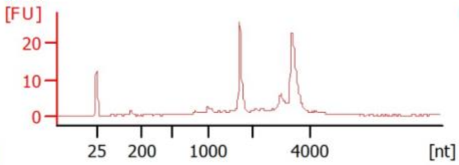

CD 293 T6
RIN: 8.70

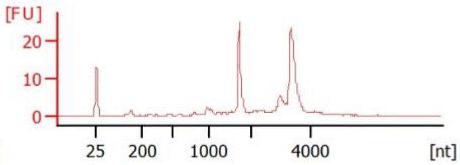

CD C22 1
RIN: 9.60

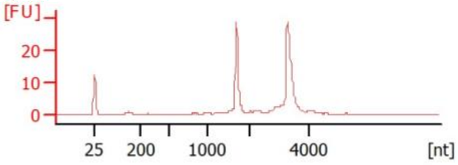

CD C22 2
RIN: 9.50

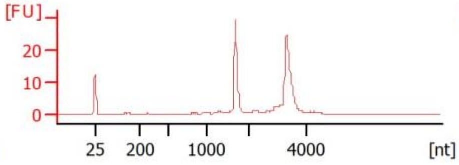

CD C22 3
RIN: 9.10

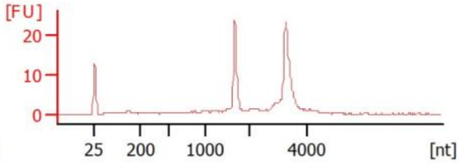

CD C22 4
RIN: 8.50

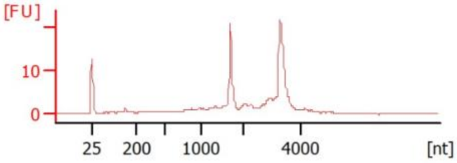

CD C22 5
RIN: 9.50

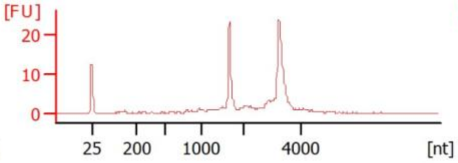

CD C22 6
RIN: 9.30

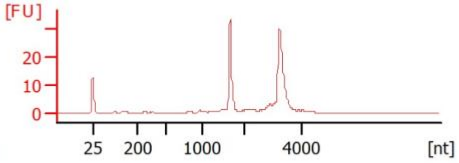

© Copyright 2003 - 2021 Agilent, Inc.

Assay Class: Eukaryote Total RNA Nano
Data Path: C:\...nalyzer\2100 expert\Data\2024-04-08\2024-04-08_13-28-24.xad
Created: 4/8/2024 1:28:23 PM
Modified: 4/8/2024 1:52:15 PM

Electropherogram Summary

CD 293 T1

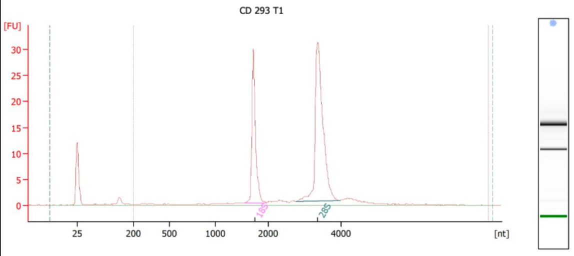

Overall Results for sample 1 : CD 293 T1

RNA Area:	131.8	Result Flagging Color:	
RNA Concentration:	85 ng/µl	Result Flagging Label:	RIN:10
rRNA Ratio [28s / 18s]:	1.9	Corr. Area 1:	126.6
RNA Integrity Number (RIN):	10 (B.02.11)		

Fragment table for sample 1 : CD 293 T1

Name	Start Size [nt]	End Size [nt]	Area	% of total Area
18S	1,551	1,975	32.7	24.8
28S	2,769	3,964	62.1	47.1

Region table for sample 1 : CD 293 T1

Name	From [nt]	To [nt]	Corr. Area	% of Total	Color
DV200	200	8,000	126.6	96	

Assay Class: Eukaryote Total RNA Nano
Data Path: C:\...nalyzer\2100 expert\Data\2024-04-08\2024-04-08_13-28-24.xad
Created: 4/8/2024 1:28:23 PM
Modified: 4/8/2024 1:52:15 PM

Electropherogram Summary Continued ...

CD 293 T2

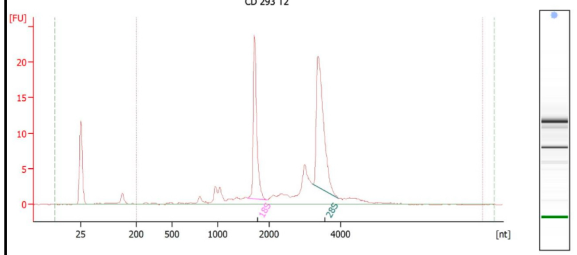

Overall Results for sample 2 : CD 293 T2

RNA Area:	121.1	Result Flagging Color:	
RNA Concentration:	76 ng/µl	Result Flagging Label:	RIN: 9.10
rRNA Ratio [28s / 18s]:	1.5	Corr. Area 1:	116.9
RNA Integrity Number (RIN):	9.1 (B.02.11)		

Fragment table for sample 2 : CD 293 T2

Name	Start Size [nt]	End Size [nt]	Area	% of total Area
18S	1,577	1,960	23.9	19.7
28S	3,202	3,939	35.5	29.3

Region table for sample 2 : CD 293 T2

Name	From [nt]	To [nt]	Corr. Area	% of Total	Color
DV200	200	8,000	116.9	96	

Assay Class: Eukaryote Total RNA Nano
Data Path: C:\...nalyzer\2100 expert\Data\2024-04-08\2024-04-08_13-28-24.xad
Created: 4/8/2024 1:28:23 PM
Modified: 4/8/2024 1:52:15 PM

Electropherogram Summary Continued ...

CD 293 T3

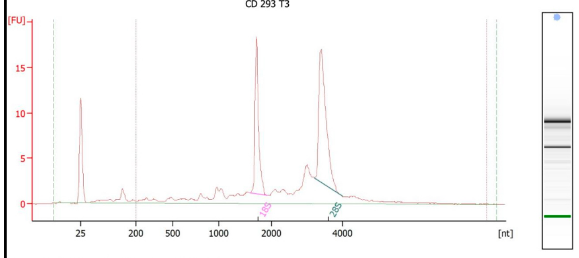

Overall Results for sample 3 : CD 293 T3

RNA Area:	121.0	Result Flagging Color:	
RNA Concentration:	76 ng/µl	Result Flagging Label:	RIN: 8.40
rRNA Ratio [28s / 18s]:	1.5	Corr. Area 1:	113.2
RNA Integrity Number (RIN):	8.4 (B.02.11)		

Fragment table for sample 3 : CD 293 T3

Name	Start Size [nt]	End Size [nt]	Area	% of total Area
18S	1,595	1,926	18.3	15.1
28S	3,213	3,985	27.8	23.0

Region table for sample 3 : CD 293 T3

Name	From [nt]	To [nt]	Corr. Area	% of Total	Color
DV200	200	8,000	113.2	94	

Assay Class: Eukaryote Total RNA Nano
Data Path: C:\...nalyzer\2100 expert\Data\2024-04-08\2024-04-08_13-28-24.xad
Created: 4/8/2024 1:28:23 PM
Modified: 4/8/2024 1:52:15 PM

Electropherogram Summary Continued ...

CD 293 T4

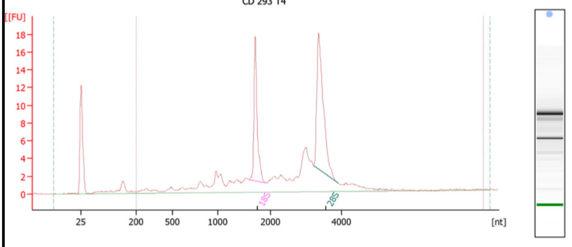

Overall Results for sample 4 : CD 293 T4

RNA Area:	122.8	Result Flagging Color:	
RNA Concentration:	77 ng/µl	Result Flagging Label:	RIN: 8.10
rRNA Ratio [28s / 18s]:	1.6	Corr. Area 1:	116.2
RNA Integrity Number (RIN):	8.1 (B.02.11, Anomaly Threshold(s) manually adapted)		

Fragment table for sample 4 : CD 293 T4

Name	Start Size [nt]	End Size [nt]	Area	% of total Area
18S	1,599	1,919	16.8	13.7
28S	3,206	3,900	26.3	21.4

Region table for sample 4 : CD 293 T4

Name	From [nt]	To [nt]	Corr. Area	% of Total	Color
DV200	200	8,000	116.2	95	

Assay Class: Eukaryote Total RNA Nano
Data Path: C:\...nalyzer\2100 expert\Data\2024-04-08\2024-04-08_13-28-24.xad
Created: 4/8/2024 1:28:23 PM
Modified: 4/8/2024 1:52:15 PM

Electropherogram Summary Continued ...

CD 293 T5

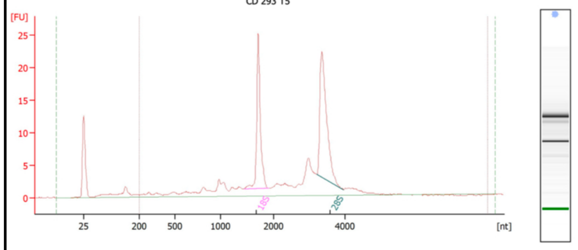

Overall Results for sample 5 : CD 293 T5

RNA Area:	152.3	Result Flagging Color:	
RNA Concentration:	95 ng/µl	Result Flagging Label:	RIN: 8.40
rRNA Ratio [28s / 18s]:	1.3	Corr. Area 1:	142.1
RNA Integrity Number (RIN):	8.4 (B.02.11)		

Fragment table for sample 5 : CD 293 T5

Name	Start Size [nt]	End Size [nt]	Area	% of total Area
18S	1,446	1,910	26.9	17.6
28S	3,221	3,965	34.3	22.5

Region table for sample 5 : CD 293 T5

Name	From [nt]	To [nt]	Corr. Area	% of Total	Color
DV200	200	8,000	142.1	93	

Assay Class: Eukaryote Total RNA Nano
Data Path: C:\...nalyzer\2100 expert\Data\2024-04-08\2024-04-08_13-28-24.xad
Created: 4/8/2024 1:28:23 PM
Modified: 4/8/2024 1:52:15 PM

Electropherogram Summary Continued ...

CD 293 T6

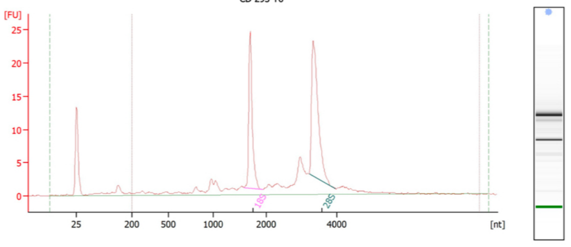

Overall Results for sample 6 : CD 293 T6

RNA Area:	137.8	Result Flagging Color:	
RNA Concentration:	86 ng/µl	Result Flagging Label:	RIN: 8.70
rRNA Ratio [28s / 18s]:	1.4	Corr. Area 1:	129.1
RNA Integrity Number (RIN):	8.7 (B.02.11)		

Fragment table for sample 6 : CD 293 T6

Name	Start Size [nt]	End Size [nt]	Area	% of total Area
18S	1,580	1,944	24.5	17.8
28S	3,210	3,944	35.4	25.7

Region table for sample 6 : CD 293 T6

Name	From [nt]	To [nt]	Corr. Area	% of Total	Color
DV200	200	8,000	129.1	94	

Assay Class: Eukaryote Total RNA Nano
Data Path: C:\...nalyzer\2100 expert\Data\2024-04-08\2024-04-08_13-28-24.xad
Created: 4/8/2024 1:28:23 PM
Modified: 4/8/2024 1:52:15 PM

Electropherogram Summary Continued ...

CD C22 1

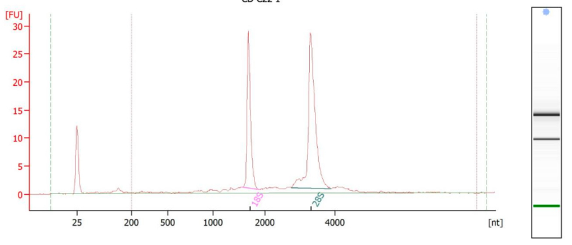

Overall Results for sample 7 : CD C22 1

RNA Area:	125.2	Result Flagging Color:	
RNA Concentration:	79 ng/µl	Result Flagging Label:	RIN: 9.60
rRNA Ratio [28s / 18s]:	1.8	Corr. Area 1:	118.6
RNA Integrity Number (RIN):	9.6 (B.02.11, Anomaly Threshold(s) manually adapted)		

Fragment table for sample 7 : CD C22 1

Name	Start Size [nt]	End Size [nt]	Area	% of total Area
18S	1,570	1,892	29.1	23.3
28S	2,764	3,873	51.5	41.1

Region table for sample 7 : CD C22 1

Name	From [nt]	To [nt]	Corr. Area	% of Total	Color
DV200	200	8,000	118.6	95	

Assay Class: Eukaryote Total RNA Nano
Data Path: C:\...nalyzer\2100 expert\Data\2024-04-08\2024-04-08_13-28-24.xad
Created: 4/8/2024 1:28:23 PM
Modified: 4/8/2024 1:52:15 PM

Electropherogram Summary Continued ...

CD C22 2

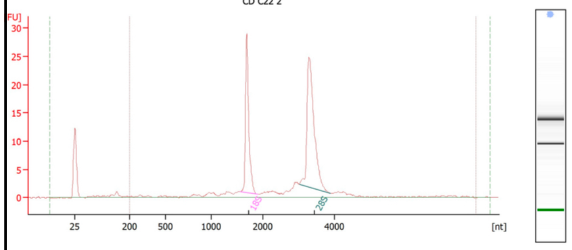

Overall Results for sample 8 : CD C22 2

RNA Area:	124.2	Result Flagging Color:	
RNA Concentration:	78 ng/µl	Result Flagging Label:	RIN: 9.50
rRNA Ratio [28s / 18s]:	1.5	Corr. Area 1:	118.0
RNA Integrity Number (RIN):	9.5 (B.02.11, Anomaly Threshold(s) manually adapted)		

Fragment table for sample 8 : CD C22 2

Name	Start Size [nt]	End Size [nt]	Area	% of total Area
18S	1,565	1,890	29.5	23.8
28S	3,018	3,896	43.4	35.0

Region table for sample 8 : CD C22 2

Name	From [nt]	To [nt]	Corr. Area	% of Total	Color
DV200	200	8,000	118.0	95	

Assay Class: Eukaryote Total RNA Nano
Data Path: C:\...nalyzer\2100 expert\Data\2024-04-08\2024-04-08_13-28-24.xad
Created: 4/8/2024 1:28:23 PM
Modified: 4/8/2024 1:52:15 PM

Electropherogram Summary Continued ...

CD C22 3

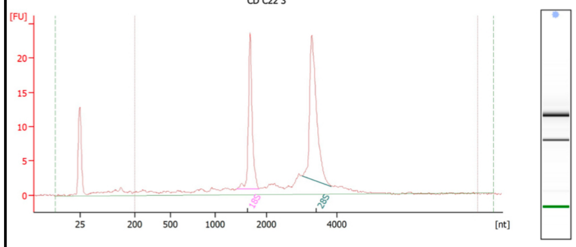

Overall Results for sample 9 : CD C22 3

RNA Area:	140.1	Result Flagging Color:	
RNA Concentration:	88 ng/µl	Result Flagging Label:	RIN: 9.10
rRNA Ratio [28s / 18s]:	1.4	Corr. Area 1:	127.8
RNA Integrity Number (RIN):	9.1 (B.02.11)		

Fragment table for sample 9 : CD C22 3

Name	Start Size [nt]	End Size [nt]	Area	% of total Area
18S	1,419	1,855	27.2	19.4
28S	3,007	3,837	39.4	28.2

Region table for sample 9 : CD C22 3

Name	From [nt]	To [nt]	Corr. Area	% of Total	Color
DV200	200	8,000	127.8	91	

Assay Class: Eukaryote Total RNA Nano
Data Path: C:\...nalyzer\2100 expert\Data\2024-04-08\2024-04-08_13-28-24.xad
Created: 4/8/2024 1:28:23 PM
Modified: 4/8/2024 1:52:15 PM

Electropherogram Summary Continued ...

CD C22 4

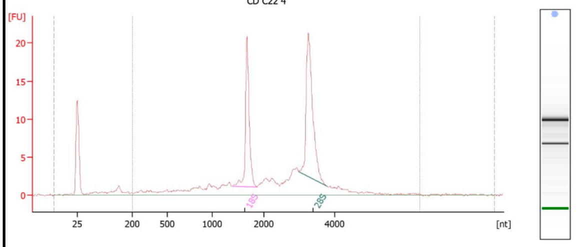

Overall Results for sample 10 : CD C22 4

RNA Area:	139.9	Result Flagging Color:	
RNA Concentration:	88 ng/µl	Result Flagging Label:	RIN: 8.50
rRNA Ratio [28s / 18s]:	1.5	Corr. Area 1:	130.4
RNA Integrity Number (RIN):	8.5 (B.02.11)		

Fragment table for sample 10 : CD C22 4

Name	Start Size [nt]	End Size [nt]	Area	% of total Area
18S	1,379	1,862	22.6	16.1
28S	2,987	3,797	32.8	23.5

Region table for sample 10 : CD C22 4

Name	From [nt]	To [nt]	Corr. Area	% of Total	Color
DV200	200	6,417	130.4	93	

Assay Class: Eukaryote Total RNA Nano
Data Path: C:\...nalyzer\2100 expert\Data\2024-04-08\2024-04-08_13-28-24.xad
Created: 4/8/2024 1:28:23 PM
Modified: 4/8/2024 1:52:15 PM

Electropherogram Summary Continued ...

CD C22 5

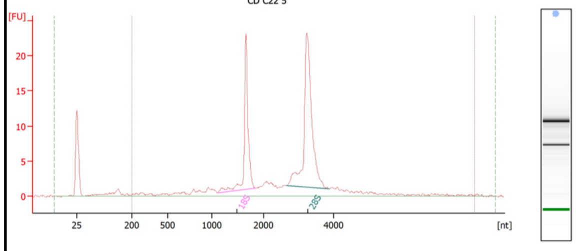

Overall Results for sample 11 : CD C22 5

RNA Area:	135.9	Result Flagging Color:	
RNA Concentration:	85 ng/µl	Result Flagging Label:	RIN: 9.50
rRNA Ratio [28s / 18s]:	1.7	Corr. Area 1:	130.0
RNA Integrity Number (RIN):	9.5 (B.02.11)		

Fragment table for sample 11 : CD C22 5

Name	Start Size [nt]	End Size [nt]	Area	% of total Area
18S	1,100	1,844	27.3	20.1
28S	2,633	3,864	46.0	33.8

Region table for sample 11 : CD C22 5

Name	From [nt]	To [nt]	Corr. Area	% of Total	Color
DV200	200	8,000	130.0	96	

Assay Class: Eukaryote Total RNA Nano
Data Path: C:\...nalyzer\2100 expert\Data\2024-04-08\2024-04-08_13-28-24.xad
Created: 4/8/2024 1:28:23 PM
Modified: 4/8/2024 1:52:15 PM

Electropherogram Summary Continued ...

CD C22 6

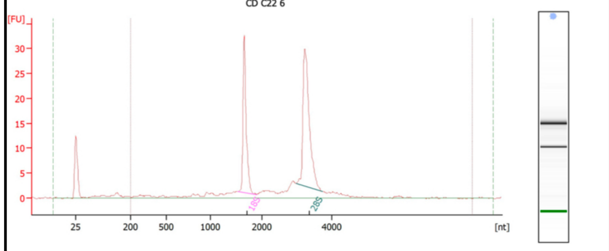

Overall Results for sample 12 : CD C22 6

RNA Area:	149.3	Result Flagging Color:	
RNA Concentration:	94 ng/µl	Result Flagging Label:	RIN: 9.30
rRNA Ratio [28s / 18s]:	1.5	Corr. Area 1:	140.6
RNA Integrity Number (RIN):	9.3 (B.02.11)		

Fragment table for sample 12 : CD C22 6

Name	Start Size [nt]	End Size [nt]	Area	% of total Area
18S	1,532	1,895	32.7	21.9
28S	2,963	3,724	48.4	32.4

Region table for sample 12 : CD C22 6

Name	From [nt]	To [nt]	Corr. Area	% of Total	Color
DV200	200	8,000	140.6	94	

SourceDataF4B

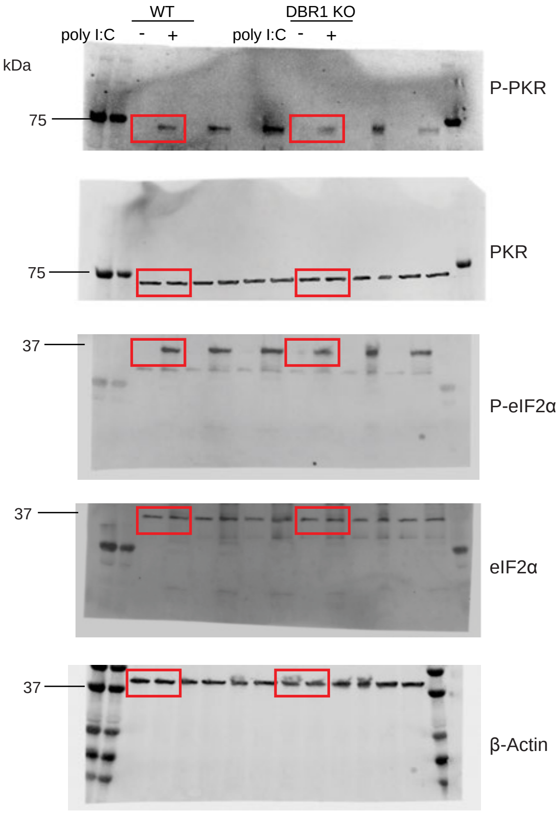

SourceDataF4C

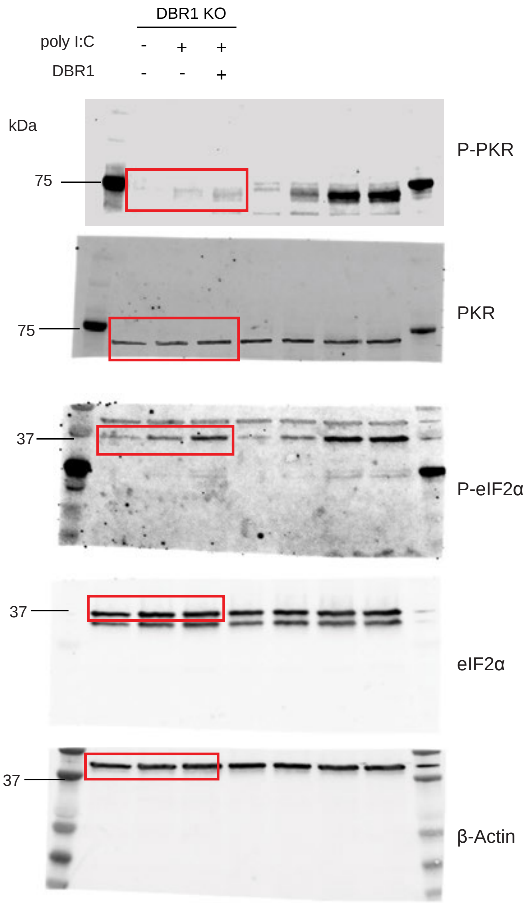

SourceDataF4D

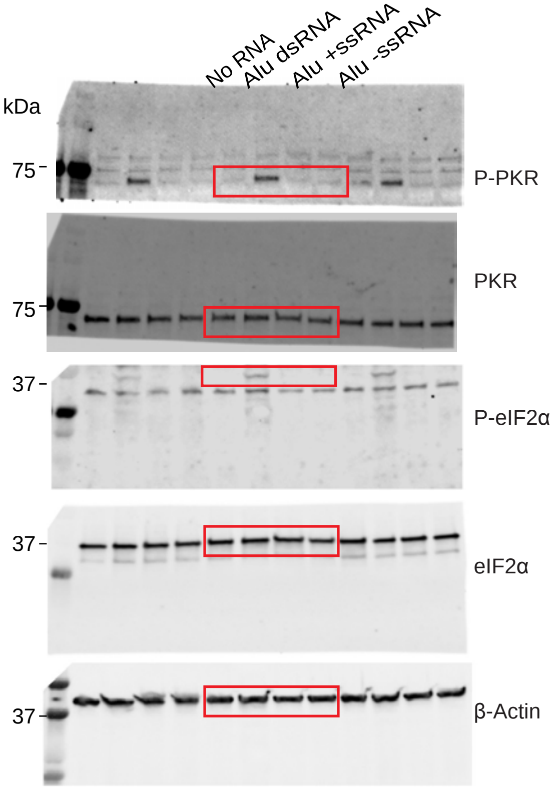

Supplement: SourceData F4 — is the source file for Fig. 4. [file jem_20250344_sourcedataf4.pdf]
