# Supplementary figures and images for: Defects in intron recycling suppress the antiviral response via a mechanism of intronic endogenous dsRNA
Source: J Exp Med. 2026 Mar 12;223(4):e20250344. doi: 10.1084/jem.20250344 (PMC13189227; doi:10.1084/jem.20250344)

SourceDataF5A

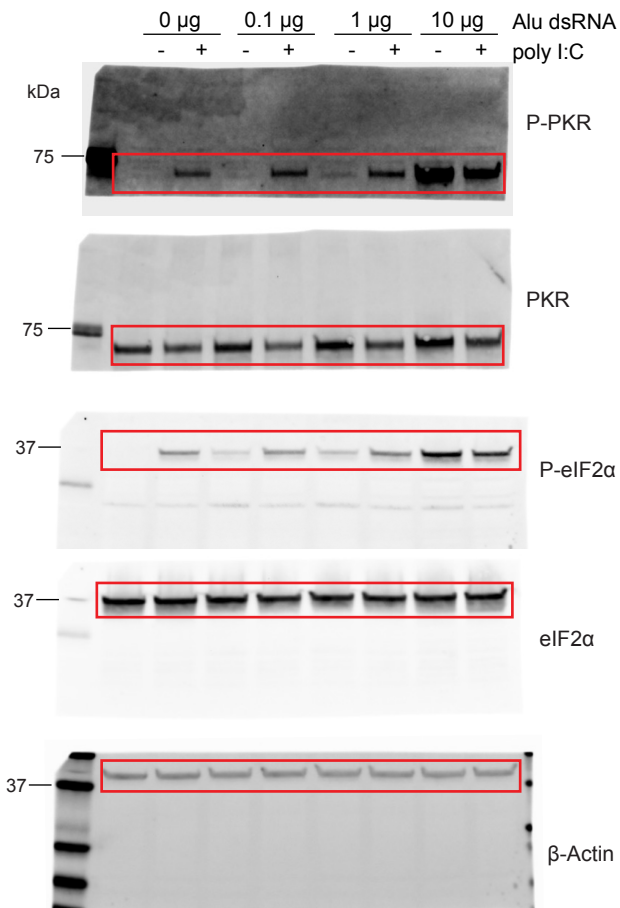

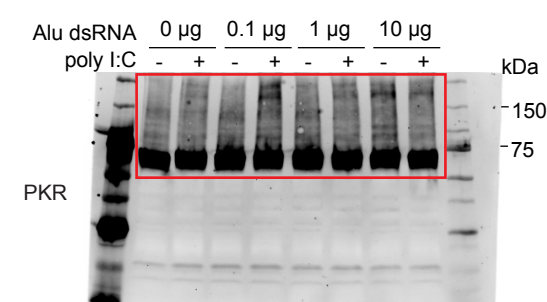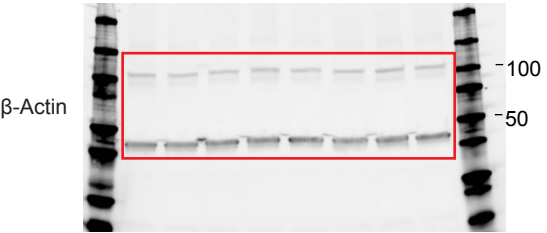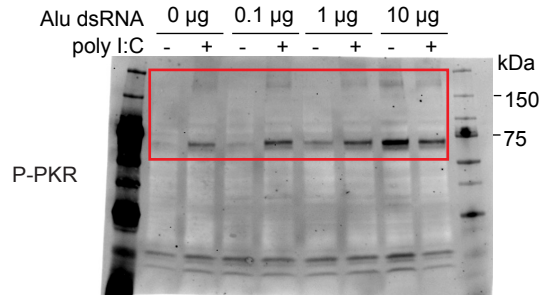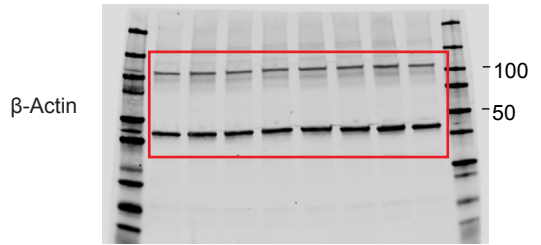

Supplement: SourceData F5 — is the source file for Fig. 5. [file jem_20250344_sourcedataf5.pdf]
