# Supplementary material for: Defects in intron recycling suppress the antiviral response via a mechanism of intronic endogenous dsRNA
Source: J Exp Med. 2026 Mar 12;223(4):e20250344. doi: 10.1084/jem.20250344 (PMC13189227; doi:10.1084/jem.20250344)
Supplement: SourceData F6 — is the source file for Fig. 6. [file jem_20250344_sourcedataf6.pdf]

SourceDataF6A

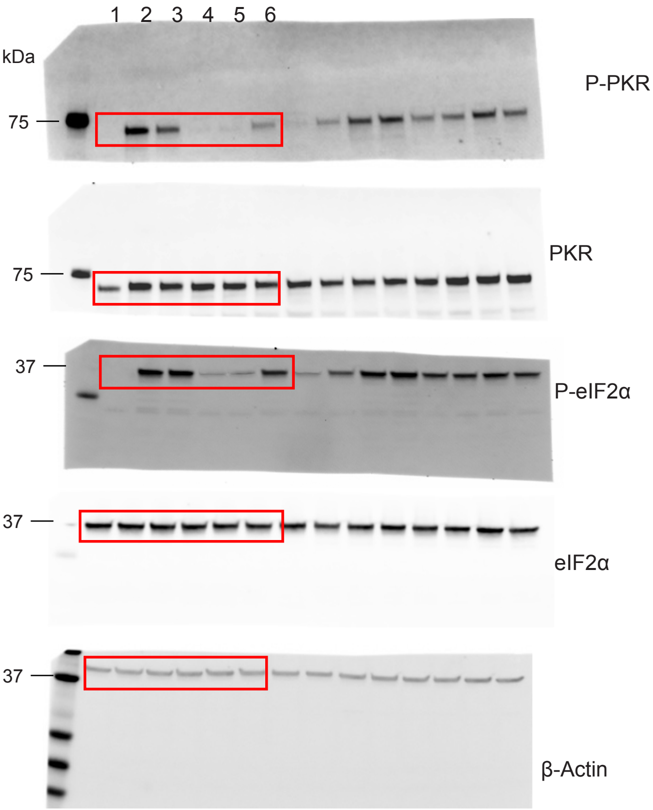

1.No RNA
2.Alu perfect dsRNA
3.Endogenous Alu dsRNA mix
4.USP8 intron 12 IR Alu
5.SNAP29 intron 1 IR Alu
6.AC009784.3 intron 1 IR Alu
