# Supplementary material for: Defects in intron recycling suppress the antiviral response via a mechanism of intronic endogenous dsRNA
Source: J Exp Med. 2026 Mar 12;223(4):e20250344. doi: 10.1084/jem.20250344 (PMC13189227; doi:10.1084/jem.20250344)

SourceDataFS3

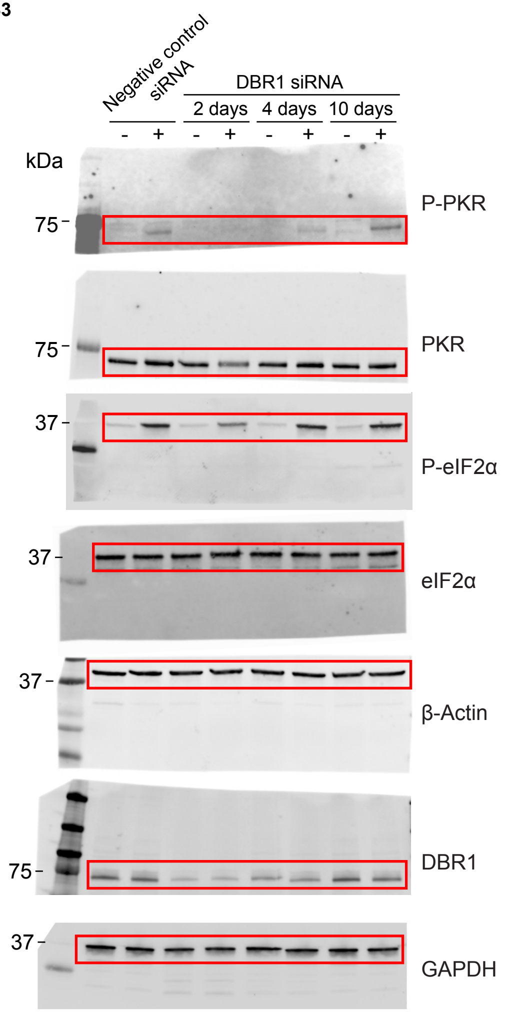

Supplement: SourceData FS3 — is the source file for Fig. S3. [file jem_20250344_sourcedatafs3.pdf]
